# Supplementary material for: Randomised, controlled, feasibility trial comparing vasopressor infusion administered via peripheral cannula versus central venous catheter for critically ill adults: A study protocol
Source: PLoS One. 2024 May 13;19(5):e0295347. doi: 10.1371/journal.pone.0295347 (PMC11090297; doi:10.1371/journal.pone.0295347)
Supplement: S1 Appendix — (PDF) [file pone.0295347.s001.pdf]

FULL STUDY TITLE

Vasopressor Infusion via Peripheral vs Central Access in patients with shock - The VIPCA randomised controlled feasibility trial.

SHORT STUDY TITLE

The VIPCA Trial

Protocol Version: 3

CONFIDENTIAL

This document is confidential and the property of the management committee.

No part of it may be transmitted, reproduced, published, or used without prior written authorisation from the institution.

STATEMENT OF COMPLIANCE

This document is a protocol for a clinical research study. The study will be conducted in compliance with all stipulations of this protocol, the conditions of ethics committee approval, the NHMRC National Statement on Ethical Conduct in Human Research (2007) and the Note for Guidance on Good Clinical Practice (CPMP/ICH-135/95).

PROTOCOL SIGNATURE PAGE

Investigator's statement

I agree to conduct this clinical investigation in accordance with the design and specific provisions of this protocol; modifications to the study are acceptable only with a mutually agreed upon amendment as approved by the Human Research Ethics Committee. I agree to await ethics approval of the protocol before initiating the study, to obtain consent from subjects as directed by the ethics committee, to collect and record data as required by the protocol and case report forms, and to maintain study documents for the period of time required.

Dr Thomas Holland

Dr Yogesh Apte

Dr Mahesh Ramanan

Ms. Stacey Watts

Dr Alison Craswell

A/Prof Frances Lin

Dr Alexis Tabah

Management Committee :

Dr Tom Holland	Chief Investigator - ED	ED Staff Specialist, Caboolture Hospital
Dr Yogesh Apte	Co-ordinating Chief Investigator - ICU	Senior ICU Staff Specialist, Caboolture Hospital
Dr Mahesh Ramanan	Chief Investigator – ICU/Supervising	ICU Staff Specialist, Caboolture Hospital
Ms. Stacey Watts	Chief Investigator - ED	ED Research Coordinator, Caboolture Hospital
Dr Alison Craswell	Associate Investigator	School of Nursing, Midwifery and Paramedicine USC
A/Prof. Frances Lin	Associate Investigator	School of Nursing, Midwifery and Paramedicine USC
Dr Alexis Tabah	Associate Investigator	ICU Staff Specialist, Redcliffe Hospital
A/Prof Chris Anstey	Associate Investigator	School of MDP Clinical Medicine, Griffith University
Prof Rob Ware	Associate Investigator	Menzies Health Institute, Griffith University
A/Prof Joshua Byrnes	Associate Investigator	Director, CAHE, School of Medicine, Griffith University

Data safety and Monitoring Committee:

An independent Data and Safety Monitoring Committee (DSMC) will monitor safety and outcome data. The DSMC will be chaired by A/Prof Kiran Shekar (Senior Intensivist and Director of Research at The Prince Charles Hospital). The other two members are Dr Antony Attokaran (Intensivist, Rockhampton Hospital) and Ms. Lauren Murray (Research Coordinator, SCUH). The DSMC will review safety data and advise the executive committee by giving recommendations on the trial continuation or aspects of the study conduct. At 50% recruitment of the planned sample (i.e., 20 patients), a formal interim analysis will be performed by the DSMC to evaluate and advise the Management Committee on continuation of the trial.

Trial registration:

This trial has been registered with the Australian New Zealand Clinical Trials Registry. ACTRN: ACTRN12621000721808p

Background	Circulatory shock affects about one-third of patients admitted to intensive care and is associated with increased mortality rates. Central venous catheters (CVCs) are commonly inserted to facilitate administration of vasopressors, but they are not without complications and pose significant logistical difficulties. There is evidence that administration of vasopressors by peripheral intravenous catheter (PIVC) has an acceptable safety profile with careful monitoring and safety precautions. The practice of commencing a vasopressor infusion via a PIVC is noted to be associated with improvements in processes of care, without increased risk of death.
Aim	The primary hypothesis is to determine whether vasopressor delivery via PIVC compared to CVC results in improved clinical outcomes, as determined by days alive and out of hospital at day 30 (DAH30).<br>The aim of this study is to test the feasibility of conducting a Phase 3 RCT using pre-defined feasibility criteria for recruitment, retention, protocol fidelity.
Design	Single centre parallel group randomised controlled feasibility trial.
Patient population	Adult patients admitted to hospital with shock needing vasopressor support.
Sample size	40 patients (20 in each group).
Methods	Eligible patients will be identified by ED or ICU staff, including medical and nursing staff, and randomised as soon as practically possible once all inclusion and exclusion criteria are satisfied. Randomisation with allocation concealment will be performed using a pre-generated randomisation sequence and sealed, opaque envelopes. Randomisation will be stratified by location of randomisation i.e., ED or ICU. Patients will be randomised to either the early CVC insertion group ('early group') or the late CVC insertion group ('late group').
Outcome measures	Primary feasibility outcome<br>• Protocol adherence (time to central line insertion in both groups; adherence to all aspects of trial protocol),<br>• Randomisation rate (target is 3 patients per month),<br>• Randomisation: Eligibility ratio (target is 0.80).<br>Primary clinical outcome<br>• Days alive and out of hospital up to day 30 (DAH30)<br>Secondary outcomes<br>• Complications related to CVC and PIVC (local, regional or systemic),<br>• Line-associate bloodstream infection,

	• Number of peripheral venous punctures and PIVC’s,<br>• Number of central lines inserted.
Study Duration	Recruitment over 1 year commencing February 2022

Glossary of Abbreviations

Abbreviation	Term
AE	Adverse Event
CLABSI	Central Line Associated Blood Stream Infection
CRF	Case Report Form
CRRT	Continuous Renal Replacement Therapy
CVC	Central Venous Catheter
DAH60	Days Alive and Out of Hospital at Day 60
DSMC	Data Safety Monitoring Committee
eCRF	Electronic Case Report Form
ED	Emergency Department
HREC	Human Research Ethics Committee
ICU	Intensive Care Unit
LOS	Length of Stay
PIVC	Peripheral Intravenous Catheter
RCT	Randomised Control Trial
REDCap	Research Electronic Data Capture
SAE	Serious Adverse Event
SPO_2	Pulse Oximeter Oxygen Saturation
VIP	Vasopressors Infusion Protocol

1. Synopsis:

Vasopressor infusions are an essential component of management of circulatory shock along with adequate fluid resuscitation, source control and appropriate and early antibiotic therapy. Vasopressor medications are generally administered centrally via a central venous catheter (CVC). The insertion of the CVC requires a trained operator, expensive equipment, USS machine, patient monitoring, a chest X-Ray for confirmation of placement etc., all of which potentially delay the administration and onset of effect of the vasopressor medications. Time is critical in circulatory shock and hence in order to minimise delay of optimal care, vasopressor medications are often initiated initially via a PIVC. The insertion of the PIVC is achieved relatively quickly, does not need specialised training, equipment or monitoring; and has relatively very few serious complications.

We hypothesize that administration of vasopressor medications via peripheral intravenous catheter over a short duration, in controlled doses and with appropriate monitoring is as safe and effective compared to that via central venous catheter in terms of patient outcomes.

This pilot phase 2 study will enrol 40 patients (20 in each group) who are admitted with shock needing vasopressor infusions. Eligible patients will be identified, and where unable to provide valid consent, enrolled utilising a waiver of consent. Patients will be randomised to two groups and further care will be delivered accordingly. Patients will be randomised to either the early CVC insertion group (CVC insertion <4 hours from randomisation, known as 'early group') or the late CVC insertion group (CVC insertion after 12 hours, known as 'late group'). The primary endpoints of the study will be feasibility of the protocol, using pre-defined feasibility criteria for recruitment, retention, and protocol fidelity.

2. Key potential benefits of this trial:

a. Improved patient comfort from not having to undergo a CVC insertion,
b. Quicker and/or sooner (or earlier) delivery of VP medications and therefore potentially equivalent outcomes such as days alive and out of hospital,
c. Provide cost savings due to use of inexpensive equipment, reduced need for staff training for insertion of CVCs etc.; and

d. Broader applicability to clinical situations in places like pre-hospital care, rural and regional hospitals, remote locations as well as low-income countries where access to early CVC insertion is difficult due to lack of trained personnel and sophisticated equipment.

3. Background and rationale:

Circulatory shock affects about one-third of patients admitted to intensive care(1) and is associated with increased mortality rates(1–3). Four pathophysiological mechanisms of shock (i.e., distributive, hypovolemic, cardiogenic, and obstructive) have been distinguished(3,4), which can be present alone or in combination(5). Vasopressor medications are utilised to restore haemodynamic stability and maintain blood pressure in patients with shock(6) from various mechanisms. Although early administration of vasopressors are significantly associated with increased shock control(7), they are not without adverse effects(8). CVCs are commonly inserted to facilitate administration of vasopressors(9) however, of those patients who receive a CVC more than 15 percent develop potentially serious complications including infectious, mechanical and thrombotic complications. The urgency to commence vasopressors via a CVC poses logistical difficulties as safe placement of a CVC requires expertise, time and resources that may be difficult to mobilise expeditiously(10).

The use of a PIVC for administration of vasopressors is recommended in patients with a contraindication to a CVC(11). The practice of commencing a vasopressor infusion via a PIVC is noted to be associated with improvements in processes of care, without increased risk of death(12). There is evidence that administration of vasopressors by PIVC has an acceptable safety profile with careful monitoring and safety precautions(13–18). Although administration of vasopressor infusion via a PIVC is not associated with increased morbidity it can lead to complications(19) such as extravasation causing skin and soft tissue necrosis and inadequate drug delivery. The current evidence on tissue injury or extravasation from vasopressor administration via PIVCs is derived mainly from case reports(20). A recent systematic review(21) reported that extravasation is uncommon and is unlikely to lead to major complications when vasopressors administered via PIVCs are given for a limited duration and under close observation.

We conducted a retrospective cohort study on vasopressor administration at Caboolture Hospital during a 12-month period. We identified 212 patients who received vasopressor infusion, 39 via peripheral only (Group 1), 155 via peripheral followed by central (Group 2) and 18 received via central only (Group 3). There were some baseline differences between groups, Group 1 had lowest median APACHE-3 score (64, IQR 44-77) and Group 3 the highest (86, IQR 57-101). Duration of vasopressor infusion too was different: Group 1 had median of 10.5, Group 2 had 18 and Group 3 25.7 hours. There were no serious complications, minor complications occurred (28% of Group 1 and 23% of Group 2 patients). Duration of peripheral vasopressor infusion was not associated with increased risk of complications. Our study found that administration of vasopressor infusions for a short duration in critically ill patients via a peripheral venous canula was occurring regularly, with low rates of complications, and offered a potentially safe alternative to central venous access. Further studies are required to test whether delivery of vasopressor infusions to critically ill patients via PIVCs has a comparable safety and efficacy profile compared to delivery via CVCs. To fill this evidence gap, we have developed the “Vasopressors Infused Peripherally (VIP)” research program.

4. Research questions:

This study protocol describes the conduct of a feasibility trial to establish and refine the plan for a Phase 3 RCT to test the hypothesis.

a. Study aims:

- To test the feasibility of conducting a Phase 3 RCT using pre-defined feasibility criteria for recruitment, retention, and protocol fidelity,
- To use feasibility data to refine the Phase 3 RCT protocol; and
- To inform sample size estimates for a Phase 3 RCT and test the data analysis plan,
- To compare the time taken before inotropes are infused
- A sub study aims to understand PIVC device selection, decision making on insertion and site management.

- To perform pre-modelling and micro-costing analyses in preparation for a health economic analysis

b. Hypothesis for the Phase 3 RCT: We hypothesise that for patients admitted into the Intensive Care Unit who need vasopressor infusions, the delivery of vasopressor initially via PIVC followed by CVC results in same or better patient outcomes than delivery via CVC as soon as possible, as determined by days alive and out of hospital at day 30 (DAH30) with an acceptable safety profile.

5. Key feasibility criteria

Feasibility studies are inappropriate for testing hypotheses in small samples(22,23), thus, the feasibility for a full trial will test the following criteria:

- Recruitment: ≥ 80% of eligible participants will be randomised; recruitment rate of at least 1 patient per week,
- Protocol fidelity: ≥ 95% of participants in each of the allocated group will receive the intervention they were allocated within the stipulated timeframes
- Retention: >95% of will consent to ongoing participation in the trial and <10% of patients will be lost to follow up; and
- Missing data: < 10%.

6. Methods:

a. Study Design

This will be a single-centre parallel-group feasibility randomised controlled trial.

b. Setting

The VIPCA trial will be conducted in the ED and ICU of Caboolture Hospital, Queensland, Australia. The Caboolture Hospital ED is a general urban district ED with 45 clinical spaces, catering for approximately 60,000 patients per annum. The Caboolture Hospital ICU is a mixed general medical-surgical-obstetric teaching unit with 4 ventilator-equivalent beds and caters for approximately 450 admissions per annum.

c. *Sample and sample size:* 40 patients (20 in each group). No formal power calculations performed as this is a feasibility trial and the superiority of one intervention over another is not being tested.

7. Study Population

Inclusion and Exclusion criteria

Inclusion criteria

- Patients admitted to Caboolture Hospital ED
- Any unplanned admission to Caboolture Hospital ICU
- ≥18 years
- Treating clinician has deemed that a VPI is required. Note - the treating clinician will assess each patient on a case by case basis and in accordance with best medical practice. The treating clinician will consider all relevant parameters including (but not limited to) blood pressure, fluid balance status and laboratory results. All aspects of the VPI infusion (dose, duration, drug used) aside from route of delivery will be determined by the treating clinician.

Exclusion criteria

- Pregnancy or suspected pregnancy,
- Treating clinician believes that survival beyond 48 hours is unlikely ***or*** patient being admitted to ICU solely for Palliation or Organ Donation
- Has received vasopressor infusion for ≥ 4 hours,
- Requiring >0.1mcg/kg/min noradrenaline (or equivalent dose of other vasopressors) at the time of screening; or requiring >1 vasopressor agent,
- Patient already has a CVC in-situ or requires a CVC insertion for specific therapies other than vasopressors (e.g., total parenteral nutrition, severe electrolyte derangements like: $K^+ \leq 2.0$ mmol/L, $PO_4^{-2} \leq 0.3$ mmol/L, or for Ca^{+2} infusion for CRRT).

8. Device selection and management sub study

A sub study will collect and analyse data for patients randomised to late group during insertion and management of their peripheral IV site. A decision-making tool has been developed based on existing evidence(24,25). When a PIVC is inserted, a member of either the study team, or another staff member trained in study procedures, will approach the operator who performed the procedure to complete a REDCap survey as soon as practicable after the insertion. The data will assist to understand health professional decision making for PIVC insertion and management for delivery of peripheral vasopressors. A hard copy of the VIPCA sub-study data collection tool has been included in Appendix A.

9. Participant enrolment and Randomisation:

Eligible patients will be identified by trained ED or ICU staff, including medical and nursing staff, using a participant screening form, and randomised as soon as practically possibly once all inclusion and exclusion criteria are satisfied. Randomisation with allocation concealment will be performed using a pre-generated randomisation sequence and sealed, opaque envelopes. Randomisation will be stratified by location of randomisation i.e., ED or ICU.

Patients will be randomised to either the early CVC insertion group ('early') or the late CVC insertion group ('late').

a. <u>Peripheral Vasopressor group *(Late Central group)* – usual care *plus*</u>

  i. PIVC, 18-gauge preferred,
  ii. Delayed insertion of CVC – A CVC is not to be inserted for at least 12 hours from randomisation,
  iii. A CVC can be inserted earlier than 12 hours if required for the following reasons:
    - Noradrenaline-equivalent dose ≥0.2mcg/kg/min,
    - Need for irritant medications/infusions that cannot be administered via a PIVC,
    - Failure of drug delivery via PIVC,
    - Complications of PIVC including extravasation of VPI, or tissue necrosis.

Where the patient is randomised to the Late Central group, the Caboolture Hospital Emergency Department ‘Peripheral Intravenous Administration of Vasoactive Medication in the ED’ Work Unit Guideline will be followed. This document is attached in Appendix B.

b. <u>Early central vasopressor group *(Early Central group)* – usual care *plus*</u>

Early insertion of central line for commencing the VPI – central line to be inserted as soon as practical, after randomisation (target time to central delivery of VP infusion is ≤4 hours from randomisation)

Usual care will be provided as per the clinical situation and according to the treating team. This care may consist of a combination of cardiopulmonary resuscitation, fluid resuscitation, vasopressors given as bolus &/or infusion, source control including surgical intervention, antibiotics as well as investigations etc. and in accordance with standard medical practice. A VPI includes any of the following medications – Noradrenaline, Adrenaline, Metaraminol, Phenylephrine and Vasopressin.

In the event of extravasation, the management will be as per the Peripheral Intravenous Administration of Vasoactive Medication in the ED Work Unit Guideline, and as follows.

Stop the infusion immediately but do not remove the PIVC. Support haemodynamics with continued VPI infusion via another PIVC, or via central or intraosseous access. Slowly aspirate residual medication from PIVC. Clean the area with an alcohol swab and mark an outline of the extravasation to provide a baseline for monitoring.

Phentolamine will be readily available in both the emergency department and intensive care unit and will be administered as per the work unit guideline included in Appendix B - (10mg/ml vial) diluted to 10mg in 10mls 0.9% Saline (1mg/ml) (maximum adult dose 10mgs)

Draw 5mls into 5ml syringe. Inject into PIVC, then remove. Do not apply pressure to the area.

Draw remaining 5mls into 1ml tuberculin syringes. Inject 0.5ml-1ml aliquots subcutaneously around leading edge of extravasation (blanching should immediately reverse)

Cardiac monitoring will continue for at least 2 hours post extravasation of the VPI, the event documented in clinical notes and recorded as an adverse event. Nursing and medical review

of the affected area will continue for 48 hours post extravasation or longer if deemed necessary by the treating clinician.

10. Participant consent

The patient presenting with shock may be critically ill and unable to provide valid consent. The person responsible for the patient may not be known, present or contactable at the time of hospital presentation. In view of this, we seek approval for enrolment without prior consent (waiver of consent). This approach is in line with the principles in paragraph 4.4.13 of the National Statement on Ethical Conduct in Human Research and is justified on the basis that the trial is comparing the effectiveness of two accepted treatment strategies (26). Enrolment will only occur if the patient's condition requires urgent treatment which cannot be delayed, the patient meets all inclusion criteria and no exclusion criteria, and it is not possible to obtain informed consent without delaying treatment. As soon as reasonably practicable following recruitment, the participant and/or the person responsible will be informed of the participant's inclusion in the trial.

A total of 3 Participant Information Sheet and Consent Forms (PICF) will be developed. One PICF for a patient completing their own consent and one PICF for the person responsible/NOK. If a waiver of consent is utilised, the participant or person responsible is consenting to the use of data already collected, not consenting to the intervention of the research. In this case, we will seek consent to continue participation in the trial. The site principal investigator, or their nominated delegate, will provide the participant with a PICF (consent to continue) once the participant is deemed to have regained capacity. This form will explain all aspects of the trial and include the option to decline or withdraw from data collection and follow up. One copy of the PICF will remain at the investigational site, another will be placed in the participant's medical record and a third copy will be given to the participant or person responsible.

10.1 Deceased patients

Participants enrolled in this study may deteriorate rapidly and unexpectedly. In the circumstance where a participant enrolled in the study dies before consent can be obtained,

we will use participants' data for the study. All attempts to contact the family and relevant circumstances prior to the death of any participants will be documented in the medical record. Without this data, the study safety data would be compromised.

10.2 Informed consent cannot be obtained from the participant or substitute decision maker

There may be a circumstance where a participant never regains competence following enrolment into the trial. In this case, an approach will be made to the Human Research Ethics Committee to request that study data may be retained and used.

11. Participant Withdrawal

An individual participant may be prematurely discontinued at the participant's or investigator's request due to screening failure, adverse event, participant is lost to follow-up, participant voluntarily withdraws, participant is withdrawn by Investigator or person responsible, and death. Withdrawal from the study will be managed by research nurses who will, where appropriate, ensure a participant withdrawal form is completed (if practicable) by the patient or person responsible. The reason for termination will be documented in study participant file and CRF. Already-accrued data, relating to participants who cease participating in this study, will be maintained as part of the study data, except where patients withdraw voluntarily. For voluntary withdrawals, all clinical data will be destroyed.

12. Patient monitoring:

All patients included in the trial will be monitored using the following:

1. Continuous ECG monitoring,
2. Non-Invasive BP monitoring to record BP at a minimum of 30-minute intervals,
3. Where possible, Arterial line with continuous invasive BP monitoring; and
4. Continuous Pulse Oximetry monitoring for SPO_2

For the two groups, there will be specific monitoring as follows:

a. ***For patients receiving peripheral vasopressor infusions:*** Monitoring for peripheral VPIs as per ED WUG (Appendix B)

b. ***For patients receiving central vasopressor infusions:*** CVC line monitoring as per current Work Unit Guidelines (WUGs) for ED & ICU (Appendix C)

Any additional monitoring will be at the discretion of the treating physician.

13. Study Outcome Measures

a. Primary feasibility outcome

- Protocol adherence (time to central line insertion in both groups; adherence to all aspects of trial protocol)
- Randomisation rate Recruitment Randomisation: Eligibility ratio
- Missing data

b. Primary clinical outcome

- Days alive and out of hospital up to day 30 (DAH30)

c. Secondary outcomes

- ICU LOS, Hospital LOS
- 30-day mortality
- Complications related to CVC and PIVC (local, regional or systemic)
- CLABSI
- Number of peripheral venous punctures
- Number of PIVCs inserted
- Number of CVCs inserted
- Healthcare costs
- Health related quality of life (PROM)
- Patient experience

14. Adverse Event Reporting:

It is recognised that the patient population in the ED and ICU will experience signs and symptoms due to the severity of underlying disease and the impact of standard treatments. These will not necessarily constitute adverse events unless they are related to study treatment or recognised to be not consistent with the patient's underlying disease and expected clinical course. According to the requirements of the National Health and Medical Research Council, Australian Health Ethics Committee Position Statement

(2009), adverse events or serious adverse events, as defined below, are not anticipated to develop as a result of study procedures.

Adverse Event (AE): any untoward medical occurrence, unintended disease or injury, or untoward clinical signs (including abnormal laboratory findings) in study participants, related to the study procedures.

Serious Adverse Event (SAE): An adverse event that led to death, or led to serious deterioration in the health of the participant, that either resulted in

a) A life-threatening illness or injury, or
b) A permanent impairment of a body structure or body function, or
c) In-patient or prolonged hospitalisation, or
d) Medical or surgical intervention to prevent life-threatening illness or injury or permanent impairment to a body structure or a body function.

14.1. Severity

The assessment of severity is a clinical determination of the intensity of an adverse event. The severity assessment for a clinical adverse event should be completed by the investigator or his/her designee using the following definitions as guidelines:

Mild: awareness of sign or symptom, but easily tolerated

Moderate: discomfort enough to cause interference with usual activity

Severe: incapacitating with inability to do work or usual activity

All AEs in this study related to VPI, PIVC and CVC, will be monitored, reported, and managed as per established unit guidelines and protocols when appropriate (Appendix A and Appendix B). These adverse events may be related to the devices or medications used as well as the existing patient condition and not related to the study. SAEs will be reported to the DSMC for review within 48 hours of occurrence. Other AEs will be notified during planned DSMC reviews. There is no specific occurrence of SAEs that define a stopping rule, and the regular review of SAEs by the DSMC will form the basis for early stopping of the study.

15. Blinding

Blinding of medical and nursing staff is not possible. The investigators will be blinded to outcome measures and primary outcomes, the data extraction from hospital databases will be conducted by a data manager who will be blinded to the randomisation, and the data analyst will be blinded.

16. Data collection

Data will be entered into dedicated electronic case report forms on electronic database REDCap®. Data pertaining to demographics, illness severity, treatment, biochemistry, clinical outcomes, and adverse events will be collected. The nested sub-study will collect data pertaining to device chosen, insertion site, attempts to gain access, total dwell time, complications, health professional rational for size, site, local anaesthetic infiltration prior to insertion, and method and quality of dressing securement. A health-related quality of life instrument (EQ-5D) will be completed at baseline and at day 30 (DAH30) follow-up. Responses will be collected from the patient directly (self-completed) or via interview with research staff via telephone. The survey will collect self-reported health-related quality of life using the EuroQol-5 Dimension, 5-level descriptive system (EQ-5D). This is a widely used preference-based instrument to measure health-related quality of life.

17. Stastical analysis

The components of feasibility will be assessed using descriptive statistics against pre-specified benchmarks. Being a feasibility trial, there will be no pre-specified thresholds of statistical significance, nor will there be any formal sample size calculations.

The primary clinical outcome of DAH-30 will be compared between the groups using an equality-of-medians test. For each treatment group, the baseline follow-up health related quality of life utility score will be estimated. The difference between the time points will be compared between each group. For all estimates, descriptive statistics (mean, standard deviation) will be provided. The responsiveness of the instrument to adverse events will be explored by comparing health related quality of life estimates between those with and those without an event of interest.

Health Economic Analysis – the primary health economic outcome measure will be the net monetary benefit of implementation. DAH-60 will be monetarised and included in the analysis using accepted threshold values for a quality adjusted life year.

Preliminary economic modelling: A probabilistic decision model will be constructed to simulate the clinical pathways associated with the two-intervention group. The preliminary model will identify all input parameters required for a full economic evaluation to be conducted alongside a fully-powered randomised control trial and determine feasibility of data collection alongside the clinical trial, as well as additional sources and reliability of estimates of the required economic input parameters. The analysis will be from a health system perspective and consider the potential cost savings from differences in utilisation of devices and consumables (including staff time associated with procedures) as well as the subsequent cost of adverse events and complications. The primary outcome measure will be the net monetary benefit of implementation. The primary trial outcome, days alive and out of hospital will be monetarised and included in the analysis using accepted threshold values for a quality adjusted life year. Resource utilisation will be collected as part of the REDCap® data eCRF and supplemented with literature searches for other model values (for example cost of adverse events). Probabilistic sensitivity analysis will be used to characterise the uncertainty in the economic evaluation based on the results of the feasibility trial. Contribution to the overall uncertainty in the economic results from each model parameter will be explored using one-way sensitivity analyses.

18. Data Management

Privacy and confidentiality of information about each participant will be maintained in all study documentation, reports and in any publications. All study information will be stored electronically on password protected files on a secure server. The information will only be made available to the Investigator team. As per the Queensland Health retention and disposal schedule, on completion of this clinical trial, any data will be retained for 25 years.

19. Ethical considerations

We will apply to The Prince Charles Hospital Human Research Ethics Committee for ethics approval with a waiver of consent.

20. Trial Governance

The chief investigators will oversee all trial procedures from development to implementation.

21. Study end point:

30 days from enrolment in this trial. There is no specific occurrence of SAEs that define a stopping rule, and the regular review of SAEs by the DSMC will form the basis for early stopping of the study.

22. Conflict of interest None.

Appendix A
VIPCA Sub Study Data Collection Tool

Appendix B
Monitoring for peripheral vasopressors – Emergency Department Work Unit Guideline

Appendix C

Central line monitoring Work Unit Guideline for ED/ICU

References

1. Sakr Y, Reinhart K, Vincent JL, Sprung CL, Moreno R, Ranieri VM, et al. Does dopamine administration in shock influence outcome? Results of the Sepsis Occurrence in Acutely Ill Patients (SOAP) Study. Crit Care Med. 2006;34(3):589–97.

2. Cecconi M, De Backer D, Antonelli M, Beale R, Bakker J, Hofer C, et al. Consensus on circulatory shock and hemodynamic monitoring. Task force of the European Society of Intensive Care Medicine. Intensive Care Med. 2014;40(12):1795–815.

3. Vincent JL, De Backer D. Circulatory shock. N Engl J Med. 2013;369(18):1726–34.

4. Weil MH. Personal commentary on the diagnosis and treatment of circulatory shock states. Curr Opin Crit Care. 2004;10(4):246–9.

5. Hiemstra B, Eck RJ, Keus F, Van Der Horst ICC. Clinical examination for diagnosing circulatory shock. Curr Opin Crit Care. 2017;23(4):293–301.

6. Hollenberg SM. Vasoactive drugs in circulatory shock. Am J Respir Crit Care Med. 2011;183(7):847–55.

7. Permpikul C, Tongyoo S, Viarasilpa T, Trainarongsakul T, Chakorn T, Udompanturak S. Early use of norepinephrine in septic shock resuscitation (CENSER) a randomized trial. Am J Respir Crit Care Med. 2019;199(9):1097–105.

8. Kwon JW, Hong MK, Park BY. Risk Factors of Vasopressor-Induced Symmetrical Peripheral Gangrene. Ann Plast Surg. 2018;80(6):622–7.

9. McGee D, Gould M. Preventing Complications of Central Venous Catheterization. N Engl J Med. 2003;348(12):1123–33.

10. Brass P, Hellmich M, Kolodziej L, Schick G, Af S, Patrick B, et al. Ultrasound guidance versus anatomical landmarks for internal jugular vein catheterization SUMMARY OF FINDINGS FOR THE MAIN COMPARISON. Cochrane Database Syst Rev. 2015;(1):CD006962.

11. Lewis T, Merchan C, Altshuler D, Papadopoulos J. Safety of the Peripheral Administration

of Vasopressor Agents. J Intensive Care Med. 2019;34(1):26–33.

12. Tian DH, Smyth C, Keijzers G, Macdonald SPJ, Peake S, Udy A, et al. Safety of peripheral administration of vasopressor medications: A systematic review. EMA - Emerg Med Australas. 2020;32(2):220–7.

13. Delgado T, Wolfe B, Davis G, Ansari S. Safety of peripheral administration of phenylephrine in a neurologic intensive care unit: A pilot study. J Crit Care [Internet]. 2016;34:107–10. Available from: http://dx.doi.org/10.1016/j.jcrc.2016.04.004

14. Cardenas-Garcia J, Schaub KF, Belchikov YG, Narasimhan M, Koenig SJ, Mayo PH. Safety of peripheral intravenous administration of vasoactive medication. J Hosp Med. 2015;10(9):581–5.

15. Pancaro C, Shah N, Pasma W, Saager L, Cassidy R, van Klei W, et al. Risk of Major Complications After Perioperative Norepinephrine Infusion Through Peripheral Intravenous Lines in a Multicenter Study. Anesth Analg. 2019;Publish Ah(Xxx):1–6.

16. Owens VS,Rosgen BK, Cherak SJ, Ferland A, Stelfox HT, Fiest KM et al. Adverse events associated with administration of vasopressor medications through a peripheral intravenous catheter: a systematic review and meta-analysis. Crit Care [internet]. 2021;25(1):1-12. Available from: http://doi.or/10.1186/s13054-021-03553-1

17. Freeth D, Gubby A, Ayida G, Berridge E, Mackintosh N, Norris B. Multidisciplinary Obstetric Simulated Emergency Scenarios (MOSES): Promoting Patient Safety in Obstetrics with Teamwork-Focused Interprofessional Simulations. J Contin Educ Health Prof. 2009;30(2):98-104.

18. Tran QK, Mester G, Bzhilyanskaya V, Afridi LZ, Andhavarapu S, Alam Z, et al. Complication of vasopressor infusion through peripheral venous catheter. A systematic review and meta-analysis. Am J Emerg Med [Internet]. 2020;38(11):2434-43. Available from http://doi.org/10.1016/j.ajem.2020.09.047

19.. Medlej K, Kazzi AA, El Hajj Chehade A, Saad Eldine M, Chami A, Bachir R, et al. Complications from Administration of Vasopressors Through Peripheral Venous Catheters:

An Observational Study. J Emerg Med [Internet]. 2018;54(1):47–53. Available from: https://doi.org/10.1016/j.jemermed.2017.09.007

20. Loubani OM, Green RS. A systematic review of extravasation and local tissue injury from administration of vasopressors through peripheral intravenous catheters and central venous catheters. J Crit Care [Internet]. 2015;30(3):653.e9-653.e17. Available from: http://dx.doi.org/10.1016/j.jcrc.2015.01.014

21. Delaney A, Finnis M, Bellomo R, Udy A, Jones D, Keijzers G, et al. Initiation of vasopressor infusions via peripheral versus central access in patients with early septic shock: A retrospective cohort study. EMA - Emerg Med Australas. 2020;32(2):210–9.

22. Thabane L, Ma J, Chu R, Cheng J, Ismaila A, Rios LP, et al. A tutorial on pilot studies: the what, why and how. BMC Med Res Methodol. 2010;10(1):1–10.

23. Leon AC, Davis LL, Kraemer HC. The role and interpretation of pilot studies in clinical research. J Psychiatr Res. 2011;45(5):626–9.

24. Larsen EN, Marsh N, O'Brien C, Monteagle E, Friese C, Rickard CM. Inherent and modifiable risk factors for peripheral venous catheter failure during cancer treatment: a prospective cohort study. Support Care Cancer. 2021;29(3):1487–96.

25. Marsh N, Webster J, Ullman AJ, Mihala G, Cooke M, Chopra V, et al. Peripheral intravenous catheter non-infectious complications in adults: A systematic review and meta-analysis. J Adv Nurs. 2020;76(12):3346–62.

26. Australian Government: National Health and Medical Research Council. Conduct in human research national statement on ethical conduct in human research. Vol. 2007. 2018. 104 p.

27. Australian Government: National Health and Medical Research Council. Conduct in human research national statement on ethical conduct in human research. Vol.2007.2018.104 p.
