# Supplementary material for: Randomised, controlled, feasibility trial comparing vasopressor infusion administered via peripheral cannula versus central venous catheter for critically ill adults: A study protocol
Source: PLoS One. 2024 May 13;19(5):e0295347. doi: 10.1371/journal.pone.0295347 (PMC11090297; doi:10.1371/journal.pone.0295347)
Supplement: S3 Appendix — (PDF) [file pone.0295347.s003.pdf]

THE COMMON GOOD
AN INITIATIVE OF THE PRINCE CHARLES HOSPITAL FOUNDATION

Grant funding agreement

Schedule

**Funder**	The Prince Charles Hospital Foundation ABN 34 853 168 302, a body corporate established by the *Hospital Foundations Act 2018* (Qld), of Level 1, Administration Building, The Prince Charles Hospital, Chermside, Queensland 4032
**Collaborator**	METRO NORTH HOSPITAL AND HEALTH SERVICE acting through Caboolture Hospital ABN 18 496 277 942 of Butterfield Street, Herston 4029
**Chief Investigator/Specified Personnel**	Dr Mahesh Ramanan
**Commencement Date**	On execution of this agreement
**Initial Period**	12 months
**Option Period**	None
**Project**	The project is described in the application CKW2022-02 titled "Vasopressor Infusion via Peripheral vs Central Access in patients with shock - The VIPCA randomised controlled feasibility trial".
**Project Plan**	The project plan is described in the application in the Project Information section.
**Research Funding**	$20,000
**Budget**	The budget is shown in the Budget section of the application.
**Funder Representative**	Dr Stephanie Yerkovich (Stephanie.Yerkovich@tpchfoundation.org.au)
**Collaborator Representative**	Angie Dobbrick (Angie.Dobbrick@health.qld.gov.au)
**Reports**	The Collaborator must submit the Reports to the Funder in the following manner:<br>(a) progress reports are required six monthly until the Project is completed, and are requested in April and October each year;<br>(b) a final report is due on the completion of the Project; and<br>(c) all reports must be submitted online.

The parties agree that the Funder is to provide funding to the Collaborator, and the Collaborator is to perform the Project on the terms attached to this Schedule.

EXECUTED as an agreement

Signed by
The Prince Charles Hospital Foundation ABN 34 853 168 302
on 30 / 08 /2022 by its duly authorised officer in the presence of:

[signature]
Signature of witness

STEPHANIE YERKOVICH
Name of witness (print)

[signature]
Signature of Authorised Officer

MICHAEL HORNBY
Full Name of Authorised Officer

CHIEF EXECUTIVE OFFICER
Office Held

Signed by
METRO NORTH HOSPITAL AND HEALTH SERVICE acting through Caboolture Hospital ABN 18 496 277 942
on 29 / 08 /2022 by its duly authorised officer in the presence of:

[signature]
Signature of witness

AMANDA SMITH
Name of witness (print)

Angie Dobbrick
Signature of Authorised Officer

ANGIE DOBBRICK
Full Name of Authorised Officer

Executive Director
Office Held

Terms

Background

A The Collaborator wishes to carry out the Project.

B The Funder agrees to provide the Research Funding to the Collaborator on the terms of this document.

Agreed terms

1 Research Funding

Funding

1.1 Subject to the Collaborator complying with this document, the Funder will grant the Research Funding to the Collaborator on the terms of this document and in the manner set out in the Schedule.

1.2 The Research Funding will be accessed by the Collaborator as follows:

(a) The Collaborator may provide internal FTE and on-cost amounts for a particular period to the Funder;

(b) the Collaborator must first obtain prior written approval from the Funder to use the Research Funding for a particular purpose;

(c) once Funder approval has been granted under clause 1.2(a), the Collaborator must purchase the approved goods or services and provide the Funder with an invoice for payment; and

(d) the Funder will pay the invoice provided under clause 1.2(c) using the Research Funds within 14 days of receiving the invoice.

1.3 The Collaborator acknowledges and agrees that, apart from the Research Funding, the Collaborator and its personnel are not entitled to any other funding, payments, sick pay or any Entitlements from the Funder.

Use of Research Funding

1.4 The Collaborator must only use the Research Funding in accordance with the Budget.

1.5 If, in the reasonable opinion of the Funder, the Collaborator has not complied with clause 1.4, the Funder may by written notice to the Collaborator:

(a) require the Collaborator to repay any Research Funding which was not spent in accordance with the Budget to the Funder; or

(b) terminate this document by written notice to the other parties,

or both.

Equipment grants

1.6 If a component of the Research Funding is allocated towards purchasing equipment for the Project:

(a) the equipment must be located at Caboolture Hospital, unless the Funder approves in writing an alternative location prior to the purchase of the equipment or until the end of the Term; and

(b) the Collaborator acknowledges the equipment is owned by the Collaborator and that the Collaborator will be responsible for all repairs, maintenance, replacements, insurances, use and warranties in respect of that equipment.

Reporting

1.7 The Funder will provide the Collaborator, Chief Investigator and any other nominated representative with a statement showing the expenditure of the Research Funding each month during the Term.

1.8 The Collaborator must, if requested by the Funder, provide the Funder within ten days of receiving a request, reasonable evidence to the Funder's satisfaction of how the Collaborator has used the Research Funding.

Additional funding

1.9 The Collaborator must immediately notify the Funder if the Collaborator receives any funding for the Project other than the Research Funding.

1.10 The Funder is not required to provide any Research Funding, and the Collaborator must not use any allocated Research Funding, for components of the Project which have been funded by third parties.

1.11 If the Funder seeks alternative or additional Research Funding for the Project, the Collaborator must provide the Funder with all reasonable assistance to try and secure the alternative or additional Research Funding.

Transfer of Research Funding

1.12 The Research Funding is not transferrable to another Collaborator without the prior written consent of the Funder.

1.13 If the Chief Investigator changes, and in the reasonable opinion of the Funder the change of Chief Investigator may result in the Project no longer being viable, the Funder may in its discretion terminate this document on 30 days' written notice to the other parties.

Suspension of funding

1.14 The Funder may suspend or terminate payment of the Research Funding under this document, or reduce the amount of the Research Funding, at any time and for any reason by written notice to the other parties, including if the Funder suspects the Collaborator of failing to comply with this document.

Dispute

1.15 If there is a dispute about whether particular Research Funding, refunds or other amounts contemplated by this document are payable or available, the Funder may withhold the amount in dispute for up to three (3) months without penalty until the resolution of the dispute.

Resources

1.16 The Collaborator must use resources efficiently when performing the Project.

2 Conduct of the Project

2.1 The Collaborator must:

(a) use the Research Funding only for the performance of this document;

(b) perform the Project in accordance with the Project Plan and Budget, and according to the terms of this document (including any applicable Guidelines);

(c) meet with the Funder to discuss the Research Funding and the Project from time to time;

(d) cooperate and provide all reasonable assistance to the Funder from time to time in respect of the Project; and

(e) deliver the Reports to the Funder as required by clause 7.

Ethics approval and clinical trials

2.2 The Collaborator must obtain all necessary ethics and clinical trial approvals required for the Project and provide evidence of such approval to the Funder before the Research Funding will be made available to the Collaborator.

2.3 For the avoidance of doubt, the Funder is not responsible or liable for securing any approvals required under clause 2.2.

Performance

2.4 The Collaborator must perform the Project in accordance with:

(a) the Project Plan, including to meet each milestone by the date specified in the Project Plan; and

(b) all applicable laws, regulations and industry standards.

2.5 The Collaborator must not vary or amend the Project Plan without the prior written approval of the Funder.

2.6 If, in the reasonable opinion of the Funder, the Project duration is expected to exceed a period of four (4) years after the Commencement Date, the Funder may terminate this document on 30 days' written notice to the other parties.

Guidelines

2.7 The Collaborator must comply with any relevant Guidelines in connection with this document.

Conflict of interest

2.8 The Collaborator represents and warrants, and it is a condition of this document, that as at the Commencement Date no conflict of interest exists, or is likely to arise, in respect of the Project.

2.9 The Collaborator must notify the Funder of any actual or potential conflict of interest which arises during the Term as soon as practicable.

2.10 On receipt of a notice under clause 2.9, the Funder will consult with the other parties and determine an appropriate course of action to effectively manage the conflict of interest.

2.11 For the avoidance of doubt, the Funder may terminate this document on 30 days' written notice to the other parties if a conflict of interest arises under clause 2.10.

3 Warranties

Intellectual Property Rights

3.1 The Collaborator represents and warrants and it is a condition of this document, that the Reports and performance of the Project will not knowingly infringe the Intellectual Property Rights or other rights of any third party.

Performance of the Project

3.2 The Collaborator represents and warrants, and it is a condition of this document that:

(a) the Collaborator has the necessary facilities, equipment, software and qualified personnel available to perform its obligations under this document;

(b) all work performed under this document will be carried out and completed efficiently, with due skill and care and to the best industry standards by qualified personnel trained and skilled in the performance of the specific services involved, in a proper and workmanlike manner and using materials suitable for the purpose;

(c) the Collaborator will hold such licences, permits, registrations and insurances as are required under any State, Territory or Commonwealth legislation or regulation to carry out any work under this document;

(d) all legal, regulatory and policy approvals (including ethics approval and any approvals in respect of conducting clinical trials) have been granted and remain in effect in respect of the Project;

(e) the Project will be performed in accordance with the Project Plan, including so as to meet each milestone specified in the Project Plan; and

(f) performance of the Project will comply with all laws.

Accuracy and reliance

3.3 The Collaborator represents and warrants and it is a condition of this document that:

(a) all information given by the Collaborator, or on Collaborator's behalf, to the Funder is complete and accurate and is not, whether by omission of information or otherwise, misleading;

(b) the Collaborator has not withheld from the Funder any document, information or other fact material to the decision of the Funder to enter into this document; and

(c) the Collaborator does not rely on any representation made to the Collaborator by the Funder or any Related Body Corporate of the Funder (if any) before entry into this document.

Acknowledgment

3.4 The Collaborator(s) each acknowledge and agree that the Funder, in entering into this document, is relying on the warranties and on the representations made in or under this document.

4 Term and termination

Term

4.1 This document commences on the Commencement Date and continues for the Initial Period and, if this document is extended under clauses 4.2 or 4.6(a), the Option Period or Extension Period (as the case may be), unless terminated earlier under clause 4.

4.2 The Funder may extend this document after the Initial Period for a period of time equal to the Option Period by giving at least three months' written notice to the other parties before the end of the Initial Period.

4.3 In the event that the Project will not be completed by the end of the Option Period, the Collaborator may provide a notice (**Extension Notice**) to the Funder as part of the Reports requesting the Term be further extended for a nominated period (**Extension Period**).

4.4 The Extension Notice must contain such information reasonably requested by the Funder from time to time to enable an assessment of the Project's viability.

4.5 The Funder may, in its discretion, accept or reject a request received under clause 4.3 on written notice to the Collaborator.

4.6 If the Funder:

(a) approves a request received under clause 4.3, this document is extended for the Extension Period; and

(b) rejects a request received under clause 4.3, this document will terminate at the end of the Option Period.

Termination for breach

4.7 A party may terminate this document by written notice to the other party if:

(a) the other party commits a material breach of this document and fails to remedy that breach within 30 days of receiving written notice from the party requiring it to do so; or

(b) an Insolvency Event occurs in relation to the other party,

in which case this document terminates immediately on giving of the notice to terminate.

After termination or expiry

4.8 On termination or expiry of this document:

(a) accrued rights or remedies of a party are not affected;

(b) subject to law, each party must return any Confidential Information or other property of the other party in that party's care, custody or control; and

(c) the Collaborator must repay any Research Funding which has not been spent or against which an expenditure has not been allocated in accordance with the Budget to the Funder.

Survival

4.9 Termination or expiry of this document will not affect clauses 1.15, **Error! Reference source not found.**, 4.8, 5, 6, 7, 8, 9, 10, 11, or any provision of this document which is expressly or by implication intended to come into force or continue on or after the termination.

5 Intellectual property

Mutual licence

5.1 Each party grants the other party an irrevocable, royalty free, transferable, sub-licensable, worldwide licence to exercise the Intellectual Property Rights in any trade mark, logo or name of the other party for the Term in connection with the Project performed under this agreement.

Ownership

5.2 The Collaborator will own all Intellectual Property Rights in the Project IP.

5.3 The Funder will own all Intellectual Property Rights in the Progress Reports.

Funder licence

5.4 The Funder grants the Collaborator an irrevocable, royalty free, non-transferable, worldwide licence to exercise the Intellectual Property Rights in the Progress Reports for non-commercial research purposes.

Commercialisation of Project IP

5.5 If the Collaborator wishes to Commercialise the Project IP, the Collaborator must first notify the Funder of its intention to do so.

5.6 The Funder may require the Collaborator to pay to the Funder, or to procure a royalty be paid to the Funder by the entity which will be Commercialising the Project IP, in recognition of the Funder providing the Collaborator with the Research Funding (**Royalty**).

5.7 The Royalty will be equal to an agreed proportion of any revenue generated from Commercialisation activities involving the Project IP, negotiated in good faith between the Collaborator and the Funder on fair and reasonable terms taking into account:

(a) the respective personnel's involvement in the creation of the Project IP;

(b) the respective cash and in-kind contributions (including the Research Funding); and

(c) other factors pertinent to the negotiations.

5.8 If the parties are unable to agree a suitable royalty under clause 5.6, the dispute must be resolved in accordance with clause 14.

6 Attribution and acknowledgment

6.1 The Collaborator must acknowledge the Funder's contribution of Research Funding in any public statement or published material (including PowerPoint presentations, flyers and event materials) relating to the Project, the Reports or any other Intellectual Property Rights created in the course of the Project.

6.2 Before disseminating any information or making any public statements under clause 6.1, the Collaborator must provide the Funder with a copy of the proposed statement.

6.3 The Funder may make press releases in relation to the Project, and use the name of the Collaborator to acknowledge their contribution to the Project.

7 Reporting and documentation

Reports

7.1 The Collaborator must provide the Funder with the Reports at the times specified in the Schedule, in a format acceptable to the Funder.

7.2 The Reports must include all information specified in the Schedule or otherwise requested by the Funder from time to time, and details of:

(a) publications relating to the Project published by the Collaborator;

(b) any funding for the Project received from a source other than the Funder;

(c) any new industry partnerships formed by the Collaborator; and

(d) any new Intellectual Property Rights generated in the course of the Project.

Additional information

7.3 If the Funder requests any additional information in relation to a Report, the Collaborator must promptly provide that information to the Funder.

8 Confidential Information

Obligations of confidence

8.1 Subject to clause 8.2, each party agrees to keep confidential, and not to use or disclose, other than as permitted by this document, any Confidential Information of the other party provided to or obtained by that party before or after entry into this document.

Exclusions

8.2 The obligations of confidence in clause 8.1 do not apply to Confidential Information:

(a) requested by the Department of Health for the State of Queensland but solely to the extent the intended disclosure is to inform that Department or the Minister for Health;

(b) that is required to be disclosed by applicable law, or under compulsion of law by a court or government agency or by the rules of any relevant stock exchange or regulator, as long as the disclosing party:

(i) discloses the minimum amount of Confidential Information required to satisfy the law or rules; and

(ii) before disclosing any information, gives a reasonable amount of notice to the other party and takes all reasonable steps (whether required by the other party or not) to maintain the Confidential Information in confidence;

(c) that is in the public domain except as a result of a breach of this document or other obligation of confidence; or

(d) that is already known by, or rightfully received, or independently developed, by the recipient of that Confidential Information free of any obligation of confidence.

Restriction on disclosure

8.3 Each party may use and disclose Confidential Information of the other party only:

(a) with the prior written consent of the other party; or

(b) to that party's directors, agents, professional advisors, employees, contractors and permitted sub-contractors solely

for the exercise of rights or the performance of obligations under this document.

8.4 If either party discloses Confidential Information under clause 8.3, that party must ensure that the information is kept confidential by the person to whom it is disclosed and is only used for the purposes of performing that party's obligations under this document.

Injunctive relief

8.5 Each party acknowledges that:

(a) the other party may suffer financial and other loss and damage if any unauthorised act occurs in relation to Confidential Information of the other party, and that monetary damages would be an insufficient remedy; and

(b) in addition to any other remedy available at law or in equity, the other party is entitled to injunctive relief to prevent a breach of, and to compel specific performance of clause 8.

9 Privacy

Treatment of Personal Information

9.1 The Collaborator shall comply with applicable Privacy Laws.

10 Indemnity

10.1 The Collaborator indemnifies the Funder against, all loss or damage incurred or suffered by the Funder arising from the negligent, unlawful or wilful acts or omissions of the Collaborator or their employees in the course of performing activities under this agreement.

(a)

10.2 The indemnity under clause 10.1:

(a) does not apply to the extent that such loss or damage is attributable to any act or omission of the Funder.

11 Insurance

11.1 Subject to clause 11.2, the Collaborator must maintain self-insurance from the Queensland Government Insurance Fund which shall be sufficient to discharge the obligation to insure.

11.2 The Collaborator shall ensure it effects and maintains workers compensation insurance as required by law.

12 No assumption of duty

The Collaborator is responsible for the discharge and satisfaction of their duties in connection with the Project and this document and neither the Funder nor any of its officers or agents assumes any duty to:

(a) advise on, supervise or control the Collaborator's performance of the Project;

(b) detect, prevent or remedy any default of the Collaborator;

(c) ensure the proper performance of any obligations of the Collaborator; or

(d) exercise any discretion for the benefit of the Collaborator.

13 Representatives

13.1 The Funder Representative will represent the Funder for the day to day purposes of this document.

13.2 The Collaborator Representative will represent the Collaborator for the day to day purposes of this document.

13.3 The Project number allocated by the Funder must be referenced on all communication between the Collaborator and the Funder.

14 Dispute resolution

Dispute

14.1 Clause 14 applies to any dispute or difference whatsoever arising out of or in connection with this document, including any question regarding its existence, validity, interpretation, breach or termination, which arises between the parties in connection with this document.

Dispute Notice

14.2 If a party considers that a dispute has arisen, it may issue a notice to the other party, setting out reasonable particulars of the matters in dispute (**Dispute Notice**).

14.3 Subject to clause 14.7, the parties must not commence or maintain any action or proceeding in any court, tribunal or otherwise regarding a dispute without first giving a Dispute Notice and complying with clause 14.

First Level Discussions

14.4 The parties must promptly hold discussions between representatives of each party after the issue of a Dispute Notice to attempt to resolve the dispute (**First Level Discussions**).

Second Level Discussions

14.5 If the dispute has not been resolved within seven days after commencement of First Level Discussions, the parties must attempt to resolve the dispute by holding discussions between a senior executive of each party (**Second Level Discussions**).

Expert determination

14.6 If the Dispute has not been resolved within ten days after commencement of Second Level Discussions, either party may refer the matter to an independent expert in accordance with, and subject to, the Expert Determination Rules.

Court proceedings

14.7 Notwithstanding anything in clause 14, a party may at any time commence court proceedings in relation to a dispute or claim arising in connection with this document where that party seeks urgent interlocutory relief.

15 Costs and taxes

Costs

15.1 Each party bears its own costs in relation to the preparation and signing of this document.

Stamp duty

15.2 The Collaborator must pay all stamp duty (including penalties and interest) assessed or payable in connection with this document.

Other taxes

15.3 Subject to clause 14, the Collaborator must pay all taxes, duties, government charges and other taxes of a similar nature (including fines, penalties and interest) imposed or levied in Australia or overseas in connection with the performance of this document.

GST

15.4 Any words capitalised in clause 15 and not already defined in clause 17.1 have the meaning given to those words in the GST Act.

15.5 Except under clause 14, the consideration for a Supply made under or in connection with this document does not include GST.

15.6 If a Supply made under or in connection with this document is a Taxable Supply, then at or before the time any part of the consideration for the Supply is payable:

(a) the Recipient must pay the Supplier an amount equal to the total GST for the Supply in addition to and in the same manner as the consideration otherwise payable under this document for that Supply); and

(b) the Supplier must give the Recipient a Tax Invoice for the Supply.

15.7 For clarity, the GST payable under clause 15.6 is correspondingly increased or decreased by any subsequent adjustment to the amount of GST for the Supply for which the Supplier is liable, however caused.

15.8 If either party has the right under this document to be reimbursed or indemnified by another party for a cost incurred in connection with this document, that reimbursement or indemnity excludes any GST component of that cost for which an Input Tax Credit may be claimed by the party being reimbursed or indemnified, or by its Representative Member, Joint Venture Operator or other similar person entitled to the Input Tax Credit (if any).

15.9 Where a Tax Invoice is given by the Supplier, the Supplier warrants that the Supply to which the Tax Invoice relates is a Taxable Supply and that it will remit the GST (as stated on the Tax Invoice) to the Australian Taxation Office.

15.10 Where a Supply made under or in connection with this document is a Progressive or Periodic Supply, clause 15.6 applies to each component of the Progressive or Periodic Supply as if it were a separate Supply.

16 General

16.1 The laws of Queensland govern this document.

16.2 Each party irrevocably submits to the non-exclusive jurisdiction of the courts of Queensland and courts competent to hear appeals from those courts.

16.3 The Collaborator must not assign, in whole or in part, or novate the Collaborator's rights and obligations under this document, without the prior consent of the Funder provided the Funder must consent to such assignments requested because of a change of machinery of government.

16.4 The Funder may assign its interest under this document.

16.5 This document supersedes all previous agreements about its subject matter. This document embodies the entire agreement between the parties.

16.6 A right under this document may only be waived in writing signed by the party granting the waiver, and is effective only to the extent specifically set out in the waiver.

16.7 This document may be signed in any number of counterparts. All counterparts together make one instrument.

17 Definitions and interpretation

Definitions

17.1 In this document the terms defined in the Schedule have the meanings given to those terms in the Schedule, and:

Bankruptcy Act means *Bankruptcy Act 1966* (Cth).

Confidential Information of a party means the terms of this document and any information:

(a) relating to the business and affairs of that party;

(b) relating to the customers, clients, employees, subcontractors or other persons doing business with that party;

(c) which is by its nature confidential;

(d) which is designated as confidential by that party; or

(e) which the other party knows or ought to know, is confidential,

and all trade secrets, knowhow, financial information and other commercially valuable information of that party, and in the case of the Funder, includes the Reports and the Research Funding.

Commercialise in relation to an Intellectual Property Right, means to manufacture, sell, hire or otherwise exploit a product or process, or to provide a service, incorporating that Intellectual Property Right, or to license, sub-license, joint venture or make any other similar arrangement with any third party to do any of those things.

Corporations Act means *Corporations Act 2001* (Cth).

Dispute means a dispute referred to in clause 14.1.

Dispute Notice means a notice referred to in clause 14.2.

Entitlements means any salary, wages, allowances, annual leave or payment in respect of annual leave, long service leave or payment in respect of long service leave, notice of termination or payment in lieu of notice of termination, severance pay, commissions, bonuses, reimbursement of expenses, superannuation and any entitlement which may be owed or payable under any award, agreement or other industrial instrument, statute or employment contract to which an employee may be entitled in respect of their employment or the termination of that employment.

Expert Determination Rules means the Resolution Institute Expert Determination Rules 2016.

Extension Notice has the meaning given to that term in clause 4.3.

Extension Period has the meaning given to that term in clause 4.3.

First Level Discussions means discussions referred to in clause 14.4.

GST Act means *A New Tax System (Goods and Services Tax) Act 1999* (Cth).

Guidelines means the instructions and guidelines published by the Funder from time to time.

Insolvency Event means any of the following events:

(f) a controller (as defined in the Corporations Act) is appointed to the party, or over any of the property of the party;

(g) the party becomes bankrupt;

(h) a controlling trustee is appointed to the party, or over any of the property of the party;

(i) the party or the party's property becomes subject to a personal insolvency arrangement under part X Bankruptcy Act or a debt agreement under part IX Bankruptcy Act;

(j) the party is unable to pay its debts when they become due and payable;

(k) the party ceases to carry on business; or

(l) any event happens in Australia or any other country or territory in respect of a party that is similar to any of the events or circumstances referred to in this definition.

Any event that takes place as part of a solvent reconstruction, amalgamation, merger, or consolidation, on terms approved in writing by the other party beforehand and in compliance with those terms is excluded from this definition.

Intellectual Property Rights means all industrial and intellectual property rights, both in Australia and throughout the world, and includes any copyright, patent, registered or unregistered trade mark, registered or unregistered design, registered or unregistered plant breeder's right, trade secret, knowhow, right in relation to semiconductors and circuit layouts, trade or business or company name, indication or source or appellation of origin or other proprietary right, or right of registration of those rights.

Material includes software, source code, object code, designs, test cases, documents, equipment, reports, technical information, customer lists, studies, plans, charts, drawings, calculations, tables, trade marks, logos, schedules and data stored by any means.

Moral Right means:

(a) the right of integrity of authorship;

(b) the right of attribution of authorship; and

(c) the right not to have authorship falsely attributed,

including those rights as conferred by the *Copyright Act 1968* (Cth), and rights of a similar nature anywhere in the world whether existing before, on or after the commencement of this document.

Personal Information has the meaning given to that term by the Privacy Act.

Privacy Laws means the *Information Privacy Act 2009* (Qld), the *Hospital and Health Boards Act 2011* (Qld) and the *Public Health Act 2005* (Qld) insofar as it may apply to the Project.

Progressive or Periodic Supply means a Taxable Supply that satisfies the requirements of section 156-5 GST Act.

Project IP means the Intellectual Property Rights created by the Collaborator in respect of the Project, excluding the Reports.

Related Body Corporate has the meaning given to that term by section 9 Corporations Act.

Royalty has the meaning given to that term in clause 5.6.

Second Level Discussions means discussions referred to in clause 14.5.

Term means the term contemplated by clause 4.1.

Interpretation

17.2 In this document:

(a) the meaning of any general language is not restricted by any accompanying example, and the words 'includes', 'including', 'such as' or 'for example' (or similar phrases) do not limit what else might be included;

(b) a reference to this document includes the agreement recorded by this document;

(c) this document is not to be interpreted against the interests of a party merely because that party proposed this document or some provision in it or because that party relies on a provision of this document to protect itself; and

(d) a reference to a party is a reference to the Collaborator or the Funder, and a reference to the parties is a reference to the Collaborator and the Funder.
