# Supplementary material for: Randomised, controlled, feasibility trial comparing vasopressor infusion administered via peripheral cannula versus central venous catheter for critically ill adults: A study protocol
Source: PLoS One. 2024 May 13;19(5):e0295347. doi: 10.1371/journal.pone.0295347 (PMC11090297; doi:10.1371/journal.pone.0295347)
Supplement: S7 Appendix — (PDF) [file pone.0295347.s007.pdf]

Work Unit Guideline

Caboolture Hospital / Emergency Department

Effective from: November 2018
Review due: November 2021

Peripheral Intravenous Administration of Vasoactive Medication in the Emergency Dept.

Peripheral Intravenous Administration of Vasoactive Medication in the Emergency Dept. **1**

Background **1**

Recent Supporting Evidence *2*

Purpose and Intent **2**

Scope and Target Audience **2**

Procedure / process **2**

Prior to Commencing Peripheral VM infusion *2*

Ensure early definitive treatment is commenced or sought, i.e.: 2

Ensure appropriate initial resuscitation and correction of fluid state. 2

Indications for peripheral administration of vasoactive medications *3*

Choice of Agent. *3*

Guidelines for pIVC used for VM infusion 3

Infusion *3*

Duration *3*

Assess for Extravasation *3*

Management of Extravasation of VM *4*

Legislation and other authority **4**

References and Benchmarking **4**

Related Documents **5**

Relevant Standards **5**

Document history **6**

AUTHORISATION **6**

Background

- Shock is a life-threatening hypo-perfusion state that requires prompt recognition and management. It remains a lethal condition with 7-day and 90-day mortality over 20% and 40% respectively.
- Timely management of septic shock has established benefits ,,. While the exact timing of when to

Queensland Government

commence vasoactive medicines is not clear, expert opinion and more recent evidence suggests increased mortality in delayed administration [illegible]

- Vasoactive Medications have traditionally only been administered via central venous lines due to concerns regarding the risk and consequences of extravasation. Difficulty or unfamiliarity with central venous access is felt to be a contributing factor to delayed administration of vasoactive agents. There is emerging evidence and experience to support the safety of pIVCs for the administration of VM, particularly if used for limited duration and with appropriate precautions [illegible]

Recent Supporting Evidence

Loubani, 2015, Systematic Review [ix];*Cardenas-Garcia*, 2015, Consecutive-patient study [vii]

- pIVC was located distal to the antecubital fossa (ACF) in 85.3% of local complications.
- Most local tissue injuries (93.5%) occurred after **> 6 hours of VM infusion**. Only 1 event was identified with infusion <1h.
- Extravasation occurred in 2% of interventions.
- **No tissue injury** at site of extravasation with timely and appropriate management.

Purpose and Intent

To provide guidance on the safe administration of vasoactive medication (VM) infusions via peripheral intravenous catheter (pIVC) for the emergent treatment of shocked adult patients in the Emergency Department.

Scope and Target Audience

Emergency Department medical, nursing and pharmacy staff.

Procedure / process

Prior to Commencing Peripheral VM infusion

Ensure early definitive treatment is commenced or sought, i.e.:

- Early Antimicrobials in septic shock
- Thrombolysis or PCI as appropriate in STEMI with cardiogenic shock.
- Haemorrhage control and timely surgical care in trauma.
- Initial IM Adrenaline in anaphylactic shock

Ensure appropriate initial resuscitation and correction of fluid state.

- 20mls/kg (up to 30mls/kg) crystalloid in septic shock; or more judicious fluids in other shock states, such as congestive cardiac failure.
- Use blood products in haemorrhagic shock;
- If critically unwell, consider:
    - Correcting Calcium (Calcium Gluconate 10% 30mls) if ionised Ca2+<1mmol/L;
    - Steroid Replacement therapy (Hydrocortisone 100mg IV TDS) for adrenal insufficiency.
    - Where IV access cannot be gained, all VM can be given via IO.

Indications for peripheral administration of vasoactive medications

- Correction of persisting hypotension in patients who have not responded to initial resuscitation.
- Haemodynamic support as a bridge to central venous access being gained.
- Haemodynamic support where duration of treatment is expected to be brief (<2h).

Choice of Agent.

- Metaraminol (0.5-1mg IV stat) may be given peripherally as a temporary measure, but should not be used in the shocked patient in lieu of vasopressors.
- Noradrenaline (6mg in 100mls 5% Glucose), commencing at 3-5mcg/min is an effective first-line agent in patients with vasodilatory (ie sepsis) and cardiogenic shock (see noradrenaline guideline).
- Adrenaline (6mg in 100mls 5% Glucose), commencing at 3-5mcg/min should be considered in patients with anaphylactic shock (see adrenaline guidelines).

Guidelines for pIVC used for VM infusion

Ideally, vein diameter >4mm measured on US and pIVC position within vein confirmed with US.
Blood return from the pIVC prior to VM administration
Contralateral to the blood pressure cuff
Do not delay based on IVC location, but aim for access away from flexor/extensor joint surfaces.
pIVC size 20 gauge or 18 gauge

Infusion

As per medication-specific guideline, via yellow-light resistant, no port access lines, with close haemodynamic monitoring.

Duration

Peripheral infusion of VM should be based on clinical situation, though prolonged peripheral VM administration should aim to be avoided. Evidence would suggest slightly increased risk for extravasation after **4-6 hours** and central access is recommended beyond this time.

Assess for Extravasation

- Monitor and document IV site
- Advise patient to notify staff of pain at administration site.
- Assessment of pIVC site and function is made with extremity checks every 30minutes for the first hour and hourly thereafter:

Early Complications	***Later Complications***
Localised pain and blanching/pallor	Erythema
Localised or distal swelling	Blistering, Skin breakdown

Increased resistance to flow or reduced flow rate	Skin/tissue Necrosis

Immediate alert by nursing staff to the medical team if extravasation signs are present, with prompt initiation of local treatment (see below).

Management of Extravasation of VM

In the event of extravasation:

1. **Stop the infusion immediately.** Do not remove the pIVC yet.
2. Support haemodynamics with continued VM infusion via another pIVC, or via Central or IO access.
3. Slowly aspirate residual medication from IVC.
4. Mark outline of the extravasation provide a baseline for monitoring. Area is cleaned with alcohol swab.
5. **Phentolamine** (10mg/ml vial)

   Diluted to 10mg in 10mls 0.9% Saline (1mg/ml)

   Draw 5mls into 5ml syringe. **Inject into pIVC, then remove**. Do not apply pressure to area

   Draw remaining 5mls into 1ml tuberculin syringes. Inject 0.5ml -1ml aliquots subcutaneously around leading edge of extravasation (Blanching should immediately reverse).

   Maximum Dose - 10mg in adults or 0.2mg/kg in children.
6. Phentolamine is a Special Access Scheme (SAS) medication. Medical officer to complete SAS Category A form and return to pharmacy (i.e. leave completed paperwork in place of removed item and notify pharmacy staff in business hours)
7. Continue cardiac monitoring for at least 2 hours post extravasation of VM.
8. Documentation in clinical notes, and medication adverse event report is completed.
9. Nursing and Medical review of affected area for at least 48 hours post extravasation of VM.

Legislation and other authority

- *Health (Drugs and Poisons) Regulation 1996*
- *Health Practitioner Regulation National Law Act 2009*

References and Benchmarking

- Holler, J et al., Shock in the emergency department; a 12 year population based cohort study; Scandinavian Journal of Trauma, Resuscitation and Emergency Medicine (2016) 24:87
- ProCESS Investigators, Yealy DM, Kellum JA, Juang DT, et al . A randomized trial of protocol - based care for early septic shock. N Engl J Med (2014); 370(18):1683-1693
- ARISE Investigators and the ANZICS Clinical Trials Group. Goal-directed resuscitation for patients with early septic shock. N Engl J Med (2014); 371:1496-1504
- Mouncey PR, Osborn TM, Power GS, et al for the ProMISe trial investigators. Trial of early, goal-directed resuscitation for septic shock. N Engl J Med (2015)

- Surviving Sepsis Campaign, Updated Bundles in Response to New Evidence, 04/2015. http://www.survivingsepsis.org/SiteCollectionDocuments/SSC_Bundle.pdf (accessed 21/11/2017)
- Beck, V et al., Timing of vasopressor initiation and mortality in septic shock: a cohort study., Critical Care (2014) 18:97
- Bai, X et al., Early versus delayed administration of norepinephrine in patients with septic shock., Critical Care (2014) 18:532
- Cardenas-Garcia et al., Safety of Peripheral Intravenous Administration of Vasoactive Medication, Journal of Hospital Medicine (Sept 2015) 10:9
- Loubani, O; Green,R; A systematic review of extravasation and local tissue injury from administration of vasopressors through peripheral intravenous catheters and central venous catheters, Journal of Critical Care (2015), 30:653–653
- Lewis, T et al., Safety of Peripheral Administration of Vasopressor Agents, Journal Of Intensive Care Medicine (2017 Jan 01), pp. 885066616686035
- Medlej, K et al., Complications from Administration of Vasopressors Through Peripheral Venous Catheters: An Observational Study, The Journal Of Emergency Medicine (2018); Vol. 54 (1), pp. 47-53

Related Documents

- Medication Administration – Nursing and Midwifery
- Medication Scope of Practice
- Medication: Adverse Drug Reactions, Allergies and Medication Alerts - Documenting, Monitoring & Communicating
- Medication: Intravenous Fluids and Medications

Relevant Standards

- National Safety and Quality Health Service Standards: Standard 3 – Preventing and Controlling Healthcare Associated Infections
- National Safety and Quality Health Service Standards: Standard 4 – Medication Safety
- National Safety and Quality Health Service Standards: Standard 6 – Communicating for Safety

Document history

**Custodian**	Senior Medical Officer, Emergency Department, Caboolture Hospital
**Risk rating**	Medium (12)
**Compliance evaluation and audit**	• Review of all reported clinical incidents regarding intravenous administration of inotropic agents via PIVC<br>• Review of PIVC insertion site post administration of IV inotropes
**Replaces Document/s**	N/A
**Document replaced**	N/A
**Key stakeholders**	Consultant Group, Emergency Department, Caboolture Hospital<br>Membership, Emergency Department SIG, Caboolture Hospital<br>NUM, Intensive Care Unit, Caboolture Hospital<br>CNC, Intensive Care Unit, Caboolture Hospital<br>Director, Intensive Care Unit, Caboolture Hospital
**Marketing Strategy**	Publication on ED SharePoint site
**Key words**	PIVC; Inotropes

AUTHORISATION

Signature Date

Chair, Emergency Department Service Improvement Group, Caboolture Hospital

Signature Date

Director, Emergency Department, Caboolture Hospital

The signed version is retained by the Service Improvement Unit.

Printed copies are uncontrolled.
