# Supplementary material for: Randomised, controlled, feasibility trial comparing vasopressor infusion administered via peripheral cannula versus central venous catheter for critically ill adults: A study protocol
Source: PLoS One. 2024 May 13;19(5):e0295347. doi: 10.1371/journal.pone.0295347 (PMC11090297; doi:10.1371/journal.pone.0295347)
Supplement: S8 Appendix — (PDF) [file pone.0295347.s008.pdf]

Caboolture Hospital Intensive Care Unit

Guideline

Effective from: February 2020
Review date: February 2023

# Central Venous Access Device (CVAD) – Insertion, Management and Removal - 005334

NSQHS STANDARDS

1. Clinical Governance
3.Healthcare-Associated Infection
5.Comprehensive Care

## Purpose and intent

This Guideline outlines the use of Central Venous Access Devices (CVAD's) primarily, Central Venous Lines (CVL), Peripherally Inserted Central Catheters (PICC) and Dialysis Access (Vascath) in the Caboolture Hospital Intensive Care Unit. It outlines the equipment required and process of clinical care to support safe insertion, the primary management requirements for safe access, and removal of CVAD's to minimise patient risk and optimise patient outcomes.

- Dialysis Access (Vascath) Catheters MUST be removed prior to transfer from ICU to a ward. The only exception to this is as per the order of an ICU consultant and a suitably accepting ward.
- CVL lines may only go to the ward at the discretion of the medical officer and the accepting medical/surgical team.
- PICC lines may be accepted by any inpatient ward.

## Scope and target audience

This Guideline applies to all Caboolture Hospital Intensive Care Unit (ICU) Clinical medical and nursing clinical staff (permanent, temporary and casual) and all organisations and individuals acting as its agents (including Visiting Medical Officers and other partners), involved in providing care and management of patients admitted to the ICU.

### Competency and education

All clinicians involved in the insertion and maintenance of CVAD's must ensure that this is within their scope of clinical practice, determined by the individual's credentials, education, training, competence and maintenance of performance at an expected level of safety and quality. Education is provided to nursing and medical staff through their unit induction process and formal education programs such as in-service and ICU Transition to Nursing Practice program, this includes supervised practice and/or simulation.



Queensland Government

# Procedure / process

The core principles of management of CVAD follow Aseptic Non-Touch Technique (ANTT) and the Queensland Health I-CARE guidelines. Personal protective equipment (PPE) including eye-protection must be worn for all procedures with risk of exposure to bodily fluids as per. Observe the five moments of hand hygiene throughout all clinical patient contact.

## Clinical Staff Roles and Responsibilities

| Staff role | Clinical responsibilities |
|---|---|
| **Medical Officer** | • Choice of insertion site and appropriate device selection as determined by the patient's condition, treatment plan, and where possible, in consultation with the patient and/or carer.<br>• Post-placement imaging request for X-ray confirmation of optimal tip placement, before CVAD is used<br>• Documentation of the anatomical location of the CVAD tip and confirmation the device is safe and appropriate to use for the purpose of treatment<br>• Documentation of the procedure including consent and any complications |
| **Nursing staff** | • Assisting the medical officer during CVAD insertion procedure<br>• Post insertion, the connection and zeroing of the any pressure transducer lines<br>• Observations and haemodynamic monitoring during and after the procedure<br>• Assessment and management of the CVAD insertion site and/or suture line<br>• Care and maintenance of associated infusion lines<br>• Dressing/site management<br>• Removal of line as clinically indicated/directed by medical officer |

### Multi-disciplinary team is responsible for:

- Daily review of necessity for CVAD – Intravascular devices should be removed when no longer clinically required

## Documentation requirements

The following must be documented in the approved ICU clinical information system (CIS):

- Line added to system with date, time and position of insertion and ceased in system when removed from patient.
- Measurement of the catheter from the exit site and monitoring for variances (shortening or lengthening) to external length of device, which if detected are reported to the medical officer for further investigation to ascertain tip location.
- Insertion site should be inspected for inflammation and any exudate from the site should be reported to the medical officer and recorded in the progress notes.
- Adequacy of the dressing should be assessed and documented daily (or more frequently), including all dressing changes.

**NOTE:** Any variance from the baseline/normal should be documented in progress notes in CIS and escalated for immediate review by medical officer.

## CVAD insertion process

CVAD's are introduced through the skin (percutaneous) into the subclavian vein, internal jugular vein or femoral vein by threading the catheter over a guide wire which is inserted through a needle (by the method known as the Seldinger technique).

- Different access sites have their own advantages and disadvantages and may be chosen depending on both operator and patient variables.
- The internal jugular vein is preferred over the subclavian vein because of a lower risk of pneumothorax.
- The catheter may remain in place for several days to several weeks.
- Standard precautions and principles of aseptic non-touch technique (ANTT) are adhered to during the insertion of CVAD and principles of ANTT are adhered to for their maintenance and removal.
- Prior to accessing a port or the connection of an infusion line, clean the needleless connector thoroughly with a single use, 70% isopropyl alcohol swab for 30 seconds and allow to air dry before accessing port.

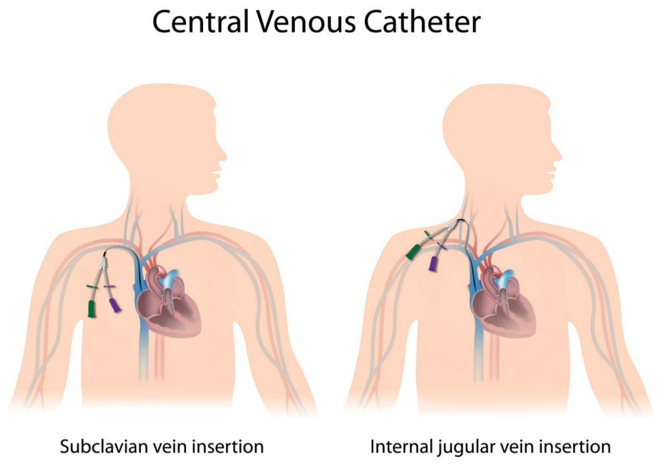


**Figure 1: CVL and Vascath insertion sites**

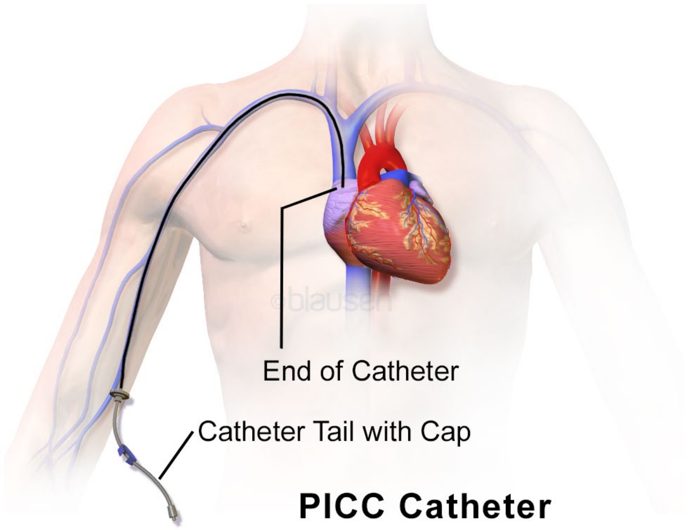


**Figure 2: PICC insertion site**

### Indications for CVAD insertion

- poor venous access
- administration of medications including vasoactive agents
- administration of IV Fluids particularly large volume replacement, and/or high regime of time critical or incompatible pharmaceuticals
- administration of Total Parenteral Nutrition (TPN)
- blood products as well as blood drawing (from selected devices)
- Continuous Renal Replacement Therapy (CRRT)
- monitor Central Venous Pressure (CVP)
  - measures Right Ventricular End Diastolic Pressure (RVEDP)
  - used as a guide to the patient's circulating volume status or the preload of the right side of the heart

## Procedural preparation for CVAD insertion

The following outlines the procedure and equipment required for insertion, maintenance and removal of CVAD's, and for CVP monitoring:

- Confirm the identity of the patient and ensure patient consent has been obtained
- It is the responsibility of the Medical Officer (MO) to ensure that the patient:
  - has had the procedure explained to them
  - has been informed of the risks associated with this procedure
  - where able, has given written consent (Central Vascular Access Device Insertion consent form)
  - has had a risk assessment completed by the MO, particularly in relation to the risk of complications, including arrhythmias, pneumothorax, air embolus, infection and haemorrhage
- Ensure patient consent has been obtained
- If the insertion of the CVAD is deemed an emergent procedure, then in the ICU there is implied consent
- The next of kin may consent for the procedure if the patient is deemed not competent to make an informed decision

### Possible complications (but not limited to):

Central venous catheterisation is an invasive technique with significant potential complications including:

- haemothorax and/or pneumothorax
- infection (systemic blood stream and local exit site infections)
- thrombosis - arterial or venous vessel injury and haematoma
- arterial puncture – inadvertent puncture of carotid, vertebral, subclavian, basilic, axillary or femoral arteries during insertion
- embolism - air or catheter-tip
- cardiac dysrhythmias and/or cardiac tamponade secondary to catheter malposition
- suboptimal placement; infiltration/extravasation of fluids into surrounding tissue or the pleural space or tissue

### General CVL product information

Multi-lumen catheter ends are colour coded, indicating the lumen size and location along the catheter i.e. distal, medial or proximal.

- The lumen ends vary in length, and it is important to check the information written on each lumen end and the main catheter stem.
- The main purpose of multi-lumen central lines is for the administration of incompatible drugs simultaneously, fluid administration and vasoactive drug administration.
- Where possible use the distal lumen for vasoactive medications or infusions.
  - Use of the 'distal lumen' will reduce the risk of inadvertent intravascular infusion and necrosis on tissue in the incidence of migration of CVAD.
- Where possible use the proximal lumen for Central Venous Pressure (CVP) monitoring.
  - Waveform tracing monitoring will alert practitioner to the malposition of the line.
  - Ideally a separate lumen is to be used for CVP monitoring.

- **Inotropes must never be coupled with other infusions.**
- Where possible use a dedicated lumen for TPN.
- **The Calcium infusion solution (pre-mixed CaCl 4.9% MgCl 3.4%) used in CRRT Citrate must be on its own lumen.**
- Aseptic non-touch technique (ANTT) must be adhered to for safe access / management of CVADs.
- The following Table contains information about the specific gauge sizes, priming volumes, flowrates of each lumen within the catheter and possible therapy combinations

## Equipment required for CVAD insertion:

- Clinicians should use sterile technique, maximal sterile barrier precautions throughout the procedure and dress in appropriate PPE
  - Sterile Gown and gloves, eye protection, face mask and hair cover
  - Drapes – Half sheet x 2, Large Fenestrated Drape x 1
- If CVP monitoring is requested
  - Disposable CVP transducer, cable and holder
  - Normal saline 500 mL and pressure bag

**NOTE:** Prime transducer set in preparation for connection prior to insertion of the CVAD as waveform analysis during insertion may assist in determining catheter position.

- Consumables:
  - Use a pre-packaged central line insertion kit if available
- If pre-packaged insertion kit not available:
  - 30 mL 70% Alcoholic Chlorhexidine (tinted pink)
  - 5 mL Lignocaine 1% (Plain; local anaesthetic)
  - Central Venous Catheterisation Set
  - Dressing pack and Suture pack
  - Ultrasound probe cover and gel (as required)
  - Disposable Luer lock syringes (2 x 5 mL, 2-3 x 10 mL)
  - Sterile saline 10mL x 3
  - Blue 23G or Green 21G Needle x 1; Red 18G Blunt Fill Needle x 2
  - Needle-free Bung connector x 4
  - Biopatch® Protective Disk
  - Silk suture - 3/0
  - Scalpel 15 blade / disposable scalpel
  - Sterile gauze squares x 4
  - Transparent semi-permeable dressing (Opsite IV3000™ or Tegaderm IV™)

## CVAD insertion procedure

**NOTE:** Ultrasound guided CVAD insertion should be strongly considered to minimise complications.

The Medical Officer inserting the CVAD is to be appropriately skilled i.e. they have been deemed capable of independent insertion after being observed by Senior Medical Staff.

1. Prepare for catheter insertion
   a. Identify appropriate insertion site and assess vein suitability
   b. Use direct visualisation technologies e.g. ultrasound guidance
2. Position patient as appropriate for insertion site:
   a. Subclavian /jugular approach: position patient head down (in slight Trendelenburg position) as tolerated or supine (if unable to tolerate head down), to reduce the risk of air embolism
   b. Femoral approach: place patient in supine position
3. Patient to be attached to a cardiac monitor during insertion
4. Arrange for administration of suitable sedation if appropriate
5. Prepare the skin with the antiseptic agent (tinted 2% Chlorhexidine) and allow to dry
6. Prepare insertion equipment and work area – **maintain a sterile field**
7. The MO performing the insertion will require full sterile precautions during insertion (head cover, face mask, gown, gloves)
8. Prime the catheter by flushing each lumen with sterile saline solution, to establish patency
9. Prepare the sterile field
10. Drape the puncture site and cover the entire patient with suitable sterile drapes
11. **During insertion, the operator and all assistants must maintain a sterile field**
12. Use local anaesthetic to prepare the site for insertion
13. Insert the CVAD and secure catheter to patient
14. Gain initial venous access
15. Insert and advance the catheter using a Seldinger technique
16. Aspirate blood to confirm intravascular position, flush each lumen and attach connectors
17. Secure catheter to patient with four site stitches using 3/0 silk
18. Caution:  minimise catheter manipulation to maintain proper catheter tip position
19. Apply a transparent, semi permeable sterile dressing to the site
20. Ensure insertion site is dry before applying dressing and apply skin protectant as needed
21. Identify the CVP transducer line: attach a blue CVL label showing “line change”
22. An X-ray is required following insertion and must be assessed by MO prior to line use

**NOTE:** The line may be used prior to X-ray in the instance where the position is confirmed with ultrasound and urgent use is indicated.

## Documentation

Documentation in the patient record is required and generally includes (but is not limited to) the following:

- Procedure specifics:
  - Informed consent (as required); inserter's identification
  - Date, time of insertion, insertion site and number of attempts
  - Elective or emergent insertion; new line or replacement
  - Use of visualization and guidance technologies e.g.USS
  - Patient position, site preparation and technique
  - Sterile field maintained throughout procedure
- Device specifics
  - Type and brand of catheter
  - Length and size of CVAD device
  - External length
- Patient assessment and response:
  - Patient assessment and pre-procedural baseline vital signs
  - Patient's response to procedure and monitor for post catheter insertion complications
  - CVP transduced after insertion (if applicable)
  - Dressing type and additional antimicrobial precautions, if any
- Visual confirmation and therapy specifics:
  - Actual tip location and confirmation for the type of use (therapy and drug administration)
  - Laboratory specimen collection, as required

# CVAD dressings

**NOTE:** All CVAD dressings must remain clean, dry and intact. Aseptic non-touch technique is to be followed when changing dressings or accessing CVADs.

- A transparent, semi-permeable, self-adhesive polyurethane dressings (with documented dressing date) is to be used on all CVAD insertion sites:
  - To allow continuous observation and monitoring of the site
  - Stabilise and secure the catheter
  - Protect the site from extrinsic contamination
- Transparent polyurethane dressings are to be changed using an aseptic non-touch technique, every seven days unless otherwise clinically indicated, including:
  - Dressing is damp, soiled or lifting
  - Significant accumulation of fluid, blood or pus
  - Evidence of inflammation or symptoms of skin reaction
- A sterile, gauze-type dressing (e.g. Primapore® or Compose®) may be used as an interim measure:
  - Until X-ray line confirmation, then replaced by a transparent dressing and Biopatch® Protective Disk as soon as possible

- If there is excessive diaphoresis, ooze or bleeding from the exit site
  - In the event of allergy or skin irritation to polyurethane type dressing
- If gauze or an alternate dressing is used in combination with a semi-permeable dressing, it shall be changed at least every 24-48 hours **OR** if damp, soiled or no longer adherent
- Biopatch® Protective Disk is an antiseptic-impregnated sponge, effective in reducing vascular catheter bacterial colonisation
  - Should be used once the CVAD has been X-rayed and placement confirmed
  - Changed weekly, with dressing change (or unless excessive soiling is evident) and use documented in the patient chart
- Tegaderm™ CHG is a Chlorhexidine Gluconate I.V securment dressing that may be used instead of a Biopatch® Protective Disk

## Dressing change equipment:

- Non-sterile gloves
- Sterile gloves
- Sterile basic dressing pack
- Sterile gauze squares (extra)
- Semi Permeable Transparent Dressing (Opsite IV3000™ or Tegaderm IV™)
- Biopatch dressing (If applicable)
- Antiseptic swab stick containing 2% Chlorhexidine Gluconate in 70% isopropyl alcohol
- If chlorhexidine is contraindicated, the following solutions may be used in this order of preference:
  - 10% Povidone-iodine, 70% isopropyl alcohol, sterile 0.9% sodium chloride

## Dressing change process:

1. Set up a procedure trolley with the listed equipment
2. Confirm the patient's identification and explain the procedure to the patient
3. Perform routine hand wash and don disposable gloves
4. Remove and discard the old dressing and gloves
5. Perform routine hand wash or hand hygiene
6. Open sterile basic dressing pack; add chosen antiseptic solution and extra sterile equipment
   a. Saline may be used to remove excess soiling or blood, prior to cleansing the catheter exit site with 70% alcoholic chlorhexidine solution
7. Position patient appropriately and request they turn their head away from the catheter site
8. Observe the catheter insertion site for signs of infection
   a. If suspected infection, swab site for micro-culture and discuss CVAD removal with the M.O
9. Check the sutures are secure and the catheter is not migrating
10. Perform clinical hand wash and don sterile gloves
11. Use ANTT to clean the exit site of the catheter in a circular motion for 30 seconds (from proximal to distal) within a radius of approximately 4cm
12. Clean the exit site at least three times using a clean swab each time, then allow to dry

13. Use ANTT to apply Biopatch® if indicated, then apply appropriate dressing over catheter exit site
14. Date the appropriate portion of the dressing and document the procedure in patient notes or CIS

### CVAD daily review and site inspection

**NOTE:** CVAD use and specific therapies are reviewed daily by the MO and if no longer clinically required, are to be removed promptly.

- The request for CVAD removal must also be documented.

- Inspect the insertion site at the start of every shift (or at each dressing change if gauze is used) for:
  - erythema
  - drainage
  - tenderness or pain
  - suture integrity and catheter position
- Site appearance should not be used as the only indicator of CVAD-related infection; e.g. local inflammation is uncommon in infection caused by coagulase negative *Staphylococci*
- The patient should also be examined for fever or other signs of sepsis:
  - tachycardia
  - hypotension
  - tachypnoea
  - elevated white cell count

## Intravenous infusions and administration set changes

**NOTE:** All CVADs must be accessed using ANTT and lumen patency assessed by an unobstructed, 'Push-Pause' pulsatile flushing technique with 0.9% sodium chloride, using a 10 mL Luer-lock syringe or a BD PosiFlush™ Syringe.

- Once a giving set or infusion line has been disconnected from the CVAD Bung connector, this must be discarded and a new giving set utilised to re-access the catheter lumen
- Prior to accessing a CVAD Bung, clean the connection thoroughly with a sterile single use 70% alcohol-impregnated swab and allow to air dry

| Infusion fluid and Administration Sets | Infusion Line Changes |
|---|---|
| Administration sets and additional attachments | Changed every 72 hours |
| All transducer sets (including pressure bag fluid) | Changed every 72 hours |
| All intravenous fluids with or without an additive | Every 24 hours (or as per the manufacturer's instructions) |
| Blood products sets | PRBC - Every 2 – 4 Units completed (maximum 12 hours)<br>Platelets / FFP - Upon completion |

| | |
|---|---|
| | Albumin - Every 12 hours |
| Lipid emulsions and set e.g. Total Parenteral Nutrition | When complete – changed at least every 24 hours |
| Propofol Infusion and set | Changed every 12 hours |
| Amiodarone Infusion and set | Changed every 12 hours |
| New Central Venous Access Device | Replace all bungs, infusion lines and infusions |

### Needle-free bung connector changes on CVAD lumen

- Needle-free bung connectors should be changed every seven days, or:
  - When there is debris or residual blood, unable to be cleared by flushing, within the bung connector
  - If the bung connector has been removed for any reason
  - If there is a break in the integrity of the connector
- Any time a needleless connector is removed from a catheter, the thread of the catheter hub should be cleaned thoroughly and vigorously for 30 seconds with a single use 2% Chlorhexidine Gluconate in 70% alcohol-impregnated swab and allowed to air dry completely prior to placing a new sterile needleless connector.

## Blood collection from CVAD

**NOTE:** Collection of blood cultures should NOT be drawn from central venous catheters.

- These specimens should be collected from peripheral veins in the first instance.

- Collection of blood for other tests is to be performed through CVADs as last option available.
  - The lumen must be flushed post-blood collection with 10-20mL 0.9% Sodium Chloride.
- Blood for coagulation profile may be collected via central venous catheters if:
  - Requested by a Medical Officer
  - Anticoagulant has not been administered through the catheter for the previous 24 hours
- Standard precautions and principles of asepsis should be maintained throughout the procedure

### CVAD blood collection equipment:

- Non-sterile gloves, apron, protective eyewear
- Sterile basic dressing pack
- 10 mL 0.9% Sodium Chloride for injection
- 10ml luer lock syringes x 2
- Sterile needle-free bung connector
- BD vacutainer
- Blood specimen tubes as required
- Laboratory request forms
- 70% Alcoholic Chlorhexidine solution (tinted pink); or antiseptic swab stick containing 2% Chlorhexidine gluconate in 70% isopropyl alcohol

### Blood collection process

1. Set up a procedure trolley with the listed equipment
2. Confirm the patient's identification and check it against the pathology request form
3. Explain the procedure and place the patient in a comfortable position for both patient and the RN
4. Turn off IV fluids and clamp lines (or close any 3-way tap), even if drawing blood from another lumen
    a. **Under no circumstances should an inotropic or vasoactive infusion be stopped or clamped for this procedure**
5. Perform routine hand wash
6. Open sterile basic dressing pack and assemble sterile equipment
7. Open the ampoule of 0.9% Sodium Chloride and place beside sterile field
8. Perform clinical hand wash and don apron, protective eyewear and non-sterile gloves
9. Draw up 10 mL of 0.9% Sodium Chloride and leave on sterile field
10. Place sterile towel under CVAD lumen end, swab bung with Alcoholic Chlorhexidine and allow to air dry
11. Apply 10 mL syringe to the slip valve bung
    a. Release clamp on catheter or turn 3-way tap "ON" to the patient
12. Withdraw 10mls of blood for discard
    a. Close clamp on catheter or turn the 3-way tap to the "OFF" position
13. Remove the syringe, set-aside for discard and apply BD Vacutainer for blood specimen collection
14. Unclamp the line, insert each blood tube until filled with required amount for specimen; mix gently
15. Clamp the line to replace BD Vacutainer with 10 mL syringe of 0.9% Sodium Chloride
    a. Release the clamp (or open the 3-way tap to the "ON" position) and gently flush the catheter with 10 mL 0.9% Sodium Chloride
16. Restart prescribed IV fluids or clamp the line after locking with 0.9% Sodium Chloride
17. Label Blood specimen tubes before leaving patient's bedside
    a. Send the blood specimen(s) to pathology with the completed request form
    b. Request form should indicate blood was drawn from a central line
18. Clean and dispose of contaminated equipment; ensure patient is left comfortable

## CVP monitoring

When CVP monitoring is requested by MO, record pressures as often as requested by MO.

- Maintain the pressure bag at 300 mmHg, to maintain patency of line.
- Perform a square wave fast flush test before obtaining a reading.
- Ensure the patient's bed is level and position the patient supine or at the consistent position documented.
    - The CVP may be reliably measured at Head-of-bed positions from 0 (flat) to 60º.
- Zero transducer as per Guideline Calibration of transducers.
- The CVP reading will be displayed on the monitor as a mean value.

### Measurement limitations

- User Error - can lead to misleading and false readings
- False readings can occur if:
  - Other infusions sharing CVP line are open
  - Cannula tip is partly occluded by thrombus or vessel wall
  - Incorrect CVP transducer placement
- CVP is not a reliable indicator of left ventricular dysfunction
- Special considerations regarding mechanically ventilated patients:
  - Contraction of abdominal muscles or high intra-abdominal pressure
  - Mechanical ventilation will increase mean intrathoracic pressure and thus increase the CVP, depending on ventilator settings

## CVAD removal process

- The Medical Officer is required to daily review the clinical need for CVAD and to document the request for CVAD removal if no longer required.
- Non-tunnelled catheters can be removed by an RN or MO assessed as competent to perform the procedure; other arrangements are required for the removal of tunnelled or implanted CVADs.

**NOTE:** If the CVAD insertion site is jugular, subclavian or upper limb, there is a risk of air embolism at the time of removal, and up to 72 hours post-removal.

- The risk of air emboli is still possible up to 72 hours post-removal, proportional to the length of time the catheter was insitu, due to the persistency of the skin tract down to the vein
- To reduce this risk when removing the CVAD, the patient must be in the supine position with the insertion site lower than the level of the heart.
  - If the patient's clinical condition precludes this positioning, this must be referred to an experienced Medical Officer
  - Ensure that an appropriately applied Semi Permeable Transparent Dressing is used to cover the exit wound on completion of the removal process
  - Ensure that the exit site dressing remain intact for this period, changing if required
- Care should be taken when removing CVAD from the internal jugular, not to put unintentional pressure on the carotid sinus, which can slow the heart rate and cause venous dilation
- Registered nurses who are competent to perform the procedure should perform the removal or arrangements made to supervise non-competent RN's.
- Prior to removal confirm the type of central line to be removed and that there is an order for removal of the central line in the patient's medical record.
- Note the patient's coagulation status, especially for large bore CVAD's such as Vascath before removing.

### Equipment for CVAD removal

- non-sterile gloves, apron, protective eyewear
- sterile gloves
- sterile basic dressing pack
- sterile gauze squares (extra)

- Semi Permeable Transparent Dressing (Opsite IV3000™ or Tegaderm IV™)
- stitch cutter and sterile scissors
- sterile specimen container (if tip is required for culture)
- completed pathology request form for catheter tip microbiology culture and sensitivity
- 70% Alcoholic Chlorhexidine solution (tinted pink); or Antiseptic Swab stick containing 2% Chlorhexidine gluconate in 70% isopropyl alcohol
- if chlorhexidine is contraindicated use 10% Povidone-iodine, 70% isopropyl alcohol, sterile 0.9% sodium chloride

## CVAD removal procedure

**NOTE:** Prior to removal confirm the type of central line to be removed and that there is an order for removal of the central line in the patient's medical record.

1. Set up a procedure trolley with the listed equipment
2. Confirm the patient's identification and explain the procedure to the patient
3. Place patient in supine (tilt head down) and request they turn their head away from the catheter site
4. Turn off all infusions running via the central line and apply clamps to central line
5. Perform routine hand wash
6. Remove and discard the old dressing and gloves
7. Perform routine hand wash or hand hygiene
8. Open sterile basic dressing pack and arrange required equipment on sterile field
    a. Saline may be used to remove excess soiling or blood, prior to cleansing the catheter exit site with 70% alcoholic chlorhexidine solution
9. Don apron, non-sterile gloves and protective eyewear
10. Remove the old dressing parallel to the skin, from distal to proximal, to prevent premature dislodgment of line then discard dressing and gloves
11. Perform clinical hand wash and don sterile gloves
12. Inspect the catheter exit site for signs of discharge and inflammation
    a. If signs of infection evident, swab site for micro-culture and report to the M.O
13. Clean the exit site with gauze squares and antiseptic solution for 30 seconds and allow to air dry
14. Cut the suture anchoring the catheter
15. Ask the patient to take a deep breath and hold momentarily (Valsalva manoeuvre) during removal, then to breathe normally when instructed
16. Remove the catheter with a slow even motion and place the catheter on the sterile field
17. Upon removal immediately place sterile gauze and apply gentle pressure over the venous site for a minimum of five minutes or until haemostasis achieved
18. Re-clean the site with gauze squares and approved antiseptic solution
19. Cover the insertion site with appropriate transparent dressing; ensure its application provides a seal
20. Inspect the catheter to confirm it is intact, **if not intact notify the Medical Officer immediately**

   a. Ensure the patient remains in the supine position
   b. Monitor patient and document a baseline set of observations
21. Medical Officer must decide on action to take to ensure the complete removal of the line
22. If the tip is required for culture, use sterile scissors cut approximately 2.5 cm off the tip of the catheter and place into a sterile specimen jar
    a. Correctly label the specimen jar before leaving the patient's bedside
    b. Send the specimen to pathology with the completed pathology request form
23. Clean and dispose of contaminated equipment appropriately; reposition patient for comfort
24. Date the appropriate portion of the site dressing and document the procedure in patient notes or CIS

## Partnering with consumers

The Patient and their carer/next of kin are to be encouraged and given the opportunity to ask questions, clarify information and identify goals of care during communication processes. Staff are responsible for providing information in a way that is understandable and that meets patient's needs and are to use perception checking techniques to ensure patient and family's understanding of discussions.

## Aboriginal and Torres Strait Islander considerations

Specific cultural implications exist for the provision of healthcare for Aboriginal and Torres Strait Islander patients and their families.

- As a matter of best practice to provide culturally capable patient care, seek cultural guidance from the local Aboriginal and Torres Strait Islander Hospital Liaison Officer, the Aboriginal and Torres Strait Islander Cultural Practice Coordinator, Health Worker, the family group or the wider community.
- Refer to the Metro North Hospital and Health Service Intranet page on Cultural Resources and Queensland Health Publication: "Aboriginal and Torres Strait Islander: Patient Care Guidelines Published by the State of Queensland (Queensland Health), May 2014.

## Legislation and other authority

- Health Act 1937
- Health Practitioner Regulation National Law Act 2009

## References


Australian Commission on Safety and Quality in Health Care, Central Line Insertion and Maintenance Guidelines ANZICs April 2012.

Australian Commission on Safety and Quality in Health Care (2010) Australian guidelines for the Prevention and Control of Infection in Healthcare

Australian Commission on Safety and Quality in Health Care, National Safety and Quality Health Service Standard. ACHS EQuIP 5, Standard 1.5.2

Centre for Disease Control Atlanta, Guidelines for the prevention of intravascular catheter-related infections 2011.

Figure 1. Retrieved on November 25th, 2019 from https://www.stepwards.com/?page_id=25126

Figure 2. Retrieved on November 25th, 2019 from https://www.physio-pedia.com/Intravenous_Lines

Gillies D, Wallen MM, Morrison AL, Rankin K, Nagy SA, O'Riordan E (2008) Optimal timing for intravenous administration set replacement (Review). Cochrane Collaboration

King Edward Memorial Hospital (2011) Parental Therapy, arterial line procedure September 2011.

Ramritu. P, Halton K, Cook. D, Whitby M & Graves. N (2007) Journal of Advanced Nursing. Catheter-related bloodstream infections in intensive care units: a systematic review with meta-analysis. 62(1), 3–21

Royal Brisbane and Womens Hospital, Policy and Procedures Manual (2010). Sansivero G. Venous Anatomy and Physiology (1998): Considerations for Vascular Access Device Placement and Function. Journal of Infusion Nursing, Sep/Oct; 21(5S): S107-S114)

The State of Queensland (Queensland Health), (2011) Insertion and Management of Percutaneous Central Venous Catheters (CVC) Guideline QH-GDL-321-6-2:2012

The State of Queensland (Queensland Health), (2011) Insertion and Management of Percutaneous Central Venous Catheters (CVC) Guideline QH-GDL-321-6-2:2012

# Related documents

## Caboolture Hospital

- 003137/PROC: Insertion and Management of Intravascular Devices Procedure

## ICU Guidelines

- 004795/Guideline Calibration of Pressure Transducers

# Appendix 1 - Arrow® Quad-Lumen CVC Specifications

| Lumen | Priming Volume | Flow Rates | Suggested Infusions |
|---|---|---|---|
| **DISTAL: 16G** (Brown) | 0.43 mL | 2700 mL/h | • Vasoactive Medications<br>• Inotropes |
| **MEDIAL 1: 14G** (Grey) | 0.54 mL | 5100 mL/h | • Blood administration<br>• High volume fluids<br>• Colloid fluid administration<br>• Drug therapy |
| **MEDIAL 2: 18G** (Blue) | 0.36 mL | 1400 mL/h | • TPN<br>• Medications |
| **PROXIMAL: 18G** (White) | 0.41 mL | 1600 mL/h | • Blood sampling<br>• Drug therapy<br>• CVP monitoring |
| NOTE: Priming volumes are approximate and are done without accessories.<br>Flow rates are done with normal saline at room temperature and represent approximate flow capabilities. | | | |

# Document history

| | |
|---|---|
| **Author** | Clinical Nurse Consultant, Caboolture Hospital ICU |
| **Custodian** | Clinical Nurse Consultant, Caboolture Hospital ICU |
| **Compliance evaluation and audit** | Adverse events relating to CVAD will be documented in the patient's medical record. All clinical incidents, near-miss events and procedural noncompliance of CVAD management are to be recorded via RiskMan and addressed by the Caboolture ICU Safety and Quality Committee. This information will be used to review practice and identify areas for future quality improvement initiatives as per MNHHS Quality Improvement Procedure (PROC089). In the absence of clinical incidents, proactive annual audit from CIS data of CVAD documentation will be conducted and presented within the schedule of Caboolture ICU Safety and Quality Committee. |
| **Replaces Document/s** | **CRICU V3.1_2015/WUG** CVAD Insertion, Management and Removal |
| **Consultation** | **Key stakeholders**<br>Director, Caboolture ICU<br>Clinical Nurse Consultant Caboolture ICU<br>Nurse Educator Caboolture – CVAD<br>Staff Specialist Intensivists, Caboolture Hospital<br>Members of Caboolture ICU Safety and Quality Committee<br>CN ICU Portfolio Holder, Standard 3: Healthcare Associated Infection |
| **Marketing Strategy** | Dissemination via email cascade to key staff groups and stakeholders; online publishing on QHEPS; ICU Staff notification as per clinical in-service/meetings |
| **Key words** | CVAD; CVC; CVL; PICC, Vascath; CVP; Central line; 005334 |

**Custodian Signature** Date

Clinical Nurse Consultant, Caboolture Intensive Care Unit, Metro North Hospital and Health Service

# AUTHORISATION

**Authorising Officer Signature** Date

Director, Caboolture Intensive Care Unit, Caboolture & Kilcoy Hospitals and Woodford Correctional Health Service, Metro North Hospital and Health Service

The original signed version is kept in file at Service Improvement Unit, Caboolture Hospital, Metro North.
