# Supplementary material for: Randomised, controlled, feasibility trial comparing vasopressor infusion administered via peripheral cannula versus central venous catheter for critically ill adults: A study protocol
Source: PLoS One. 2024 May 13;19(5):e0295347. doi: 10.1371/journal.pone.0295347 (PMC11090297; doi:10.1371/journal.pone.0295347)
Supplement: S2 File — (DOCX) [file pone.0295347.s010.docx]

**S1 Appendix. VIPCA Study Protocol**. This is the main study protocol for the VIPCA trial.

**S2 Appendix. Ethics approval**. This is the ethical approval for the VIPCA trial.

**S3 Appendix. Grant Funding Agreement**. This is the confirmation of the grant received for the VIPCA trial from The Common Good Foundation.

**S4 Appendix**. **Grant Funding Letter**. This is the confirmation of the grant received for the VIPCA trial from the Emergency Medicine Foundation.

**S5 Appendix. Human Participants Research Checklist**. This is the PLOS ONE checklist for human participants in research.

**S6 Appendix. SPIRIT Checklist**. This is the SPIRIT checklist for the VIPCA Trial.

**S7 Appendix. Guideline for PIVC administration of vasoactive medication**. This is the Caboolture Hospital Emergency Department’s unit guidelines for the management of vasoactive medications administered via peripheral intravenous cannulae.

**S8 Appendix. Central Venous Access Device- Insertion, management and removal**. This is the Caboolture Hospital ICU’s guidelines for the use of central venous catheters.
