# Supplementary material for: Randomised, controlled, feasibility trial comparing vasopressor infusion administered via peripheral cannula versus central venous catheter for critically ill adults: A study protocol
Source: PLoS One. 2024 May 13;19(5):e0295347. doi: 10.1371/journal.pone.0295347 (PMC11090297; doi:10.1371/journal.pone.0295347)
Supplement: S2 Appendix — (PDF) [file pone.0295347.s002.pdf]

Queensland Government

The Prince Charles Hospital
Human Research Ethics Committee

Metro North
Hospital and Health Service

Enquiries to:	Research Ethics & Governance Unit
Telephone:	07 3139 4198
Email:	ResearchTPCH@health.qld.gov.au

27 August 2021

Dr Yogesh Apte
Caboolture Hospital

Dear Dr Apte

Re: Project ID 74377: Vasopressor Infusion via Peripheral versus Central Access in patients with shock (The VIPCA Trial) - A randomised controlled feasibility trial

Thank you for submitting the above research project for single ethical review. This project was considered by The Prince Charles Hospital Human Research Ethics Committee (TPCH HREC) **(EC00168)** at its meeting held on 26 August 2021.

I am pleased to advise that this research project meets the requirements of the National Health and Medical Research Council's (NHMRC) *National Statement on Ethical Conduct in Human Research (2007)* and that the TPCH Human Research Ethics Committee has granted final ethical approval of this research project.

Note: If additional sites are engaged prior to the commencement of, or during the research project, the Coordinating Principal Investigator is required to notify the HREC. Notification of withdrawn sites should also be provided to the HREC in a timely fashion.

This letter constitutes ethical approval only. This project cannot proceed at any site until separate research governance authorisation has been obtained from the CEO or Delegate of the institution under whose auspices the research will be conducted at that site.

The approved documents include:

Document	Version	Date
HREC/2021/QPCH/74377	Jul ver 2	
Protocol	2.0	6 July 2021
Participant Information Sheet and Consent Form: Self	2	16 April 2021
Participant Information Sheet and Consent Form: Person Responsible	1	14 July 2021
Participant Information Sheet and Consent Form: Consent to Continue	1	14 July 2021
Sub-study Data Collection Tool	1	24 April 2021
Appendix C: CVAD Guideline		February 2020

Appendix B: Peripheral Intravenous Administration Guideline		November 2018

Ongoing approval is for the duration of the project, conditional on:

1. In accordance with Section 5.5.6 (b) of the National Statement the Principal Investigator will report to the HREC annually (**Due by 30 April each year)** in the specified format and a final report is to be submitted on completion of the study. www.health.qld.gov.au/metronorth/research/ethics-governance/post-approval-reporting/default.asp

2. The Principal Investigator will immediately report anything which might warrant review of ethical approval of the project in the specified format, including:

    a. Unforeseen events that might affect continued ethical acceptability of the project.

    b. Serious Adverse Events that materially impact on the continued ethical acceptability of the project.

3. Amendments to the research project are to be submitted to the HREC for review. Amendments should be reflected in a cover letter from the principal investigator, providing a description of the changes, the rationale for the changes, and their implications for the ongoing conduct of the study. Further advice on submitting amendments is available from:

    www.health.qld.gov.au metronorth.health.qld.gov.au/research/ethics-and-governance/post-approval-reporting

4. The Principal Investigator will notify the HREC if the project is discontinued at the participating site(s) with reasons provided.

5. If additional sites are engaged prior to the commencement of, or during the research project, the Principal Investigator is required to notify the HREC. Notification of the withdrawn sites should also be provided to the HREC in a timely fashion accompanying a close out report for that site.

- **Please advise the HREC of the date you commence the research project for the approved site(s) using the Notification of Commencement Form:** www.health.qld.gov.au/metronorth/research/ethics-governance/post-approval-reporting/default.asp

- The HREC's Terms of Reference, Standard Operating Procedures, membership and standard forms are available from: https://www.health.qld.gov.au/hiiro/html/regu/regu_home

The nominated participating sites for this project are:

No.	Site
1.	Caboolture Hospital

The Prince Charles Hospital Human Research Ethics Committee wishes you every success in your research.

Yours sincerely,

Dr Russell Denman
The Prince Charles Hospital Human Research Ethics Committee
Metro North Hospital and Health Service

This HREC is constituted and operates in accordance with the National Health and Medical Research Council's (NHMRC) *National Statement on Ethical Conduct in Human Research (2007).* The processes used by this HREC to review multi-centre research proposals have been certified by the National Health and Medical Research Council.
Please be advised that in the instance of an investigator being a member of the TPCH HREC, they are absented from the decision making process relating to that study.
