# Supplementary material for: Randomised, controlled, feasibility trial comparing vasopressor infusion administered via peripheral cannula versus central venous catheter for critically ill adults: A study protocol
Source: PLoS One. 2024 May 13;19(5):e0295347. doi: 10.1371/journal.pone.0295347 (PMC11090297; doi:10.1371/journal.pone.0295347)
Supplement: S4 Appendix — (PDF) [file pone.0295347.s004.pdf]

Emergency Medicine Foundation

Suite 1b, Terraces
19 Lang Parade, Milton QLD 4064
P +61 7 3720 5700
E info@emfoundation.org.au
ACN 128 057170 ABN 17 128 057 170

Ref: 01331/22 ANV:dt

23rd August 2022

Dr Thomas Holland
Emergency Department
Caboolture Hospital
CABOOLTURE QLD 4510

Email: thomas.holland@health.qld.gov.au
stacey.watts@health.qld.gov.au
MetroNorthResearch-RGO@health.qld.vo.au

Dear Dr Holland

Re: **EMJS-411R37-2022-HOLLAND**
Title: **Vasopressor Infusion via Peripheral vs Central Access in emergency department patients with shock – The VIPCA RCT**

Thank you for your recent application to the Emergency Medicine Foundation (EMF) Queensland Research Program for funding in Grant Round 37.

Congratulations, the EMF Board wishes to advise that you have been successful in your application for the sum of **$38,766** (+GST) over a period of one (1) year under the condition that you will lead the project as Principal Investigator (PI). Dr Ramanan does not meet EMF's PI eligibility criteria.

To enable EMF to formally progress your grant, please confirm in writing by **31 August 2022** if you agree to accept this funding offer. Once we have received your acceptance, EMF will issue a funding agreement. Please note that EMF will only execute the funding agreement when written confirmation of your ethics approval has been received. Under the EMF Queensland Research Program guidelines, the offer of funding will be rescinded if the funding agreement is not executed within twelve (12) months from the date of this letter.

EMF's Grant Awards Ceremony will be held at Victoria Park on **8 March 2023**. Please ensure your availability for this event to accept the grant. Formal invitations will follow shortly.

Thank you for your interest in emergency medicine research. EMF is committed to building the capabilities of the next generation of emergency medicine researchers and I look forward to working with you on achieving your future research endeavours.

Yours sincerely

Dr Angie Nguyen Vu
Research Manager
